# Comparative Gene Expression Analysis by a Differential Clustering Approach: Application to the *Candida albicans* Transcription Program

**DOI:** 10.1371/journal.pgen.0010039

**Published:** 2005-09-30

**Authors:** Jan Ihmels, Sven Bergmann, Judith Berman, Naama Barkai

**Affiliations:** 1 Departments of Molecular Genetics and Physics of Complex Systems, Weizmann Institute of Science, Rehovot, Israel; 2 Department of Medical Genetics, University of Lausanne, Switzerland; 3 Departments of Genetics, Cell Biology & Development, and Microbiology, University of Minnesota, Minneapolis, Minnesota, United States of America; Princeton University, United States of America

## Abstract

Differences in gene expression underlie many of the phenotypic variations between related organisms, yet approaches to characterize such differences on a genome-wide scale are not well developed. Here, we introduce the “differential clustering algorithm” for revealing conserved and diverged co-expression patterns. Our approach is applied at different levels of organization, ranging from pair-wise correlations within specific groups of functionally linked genes, to higher-order correlations between such groups. Using the differential clustering algorithm, we systematically compared the transcription program of the fungal pathogen *Candida albicans* with that of the model organism *Saccharomyces cerevisiae.* Many of the identified differences are related to the differential requirement for mitochondrial function in the two yeasts. Distinct regulation patterns of cell cycle genes and of amino acid metabolic genes were also revealed and, in some cases, could be linked to the differential appearance of *cis-*regulatory elements in the gene promoter regions. Our study provides a comprehensive framework for comparative gene expression analysis and a rich source of hypotheses for uncharacterized open reading frames and putative *cis-*regulatory elements in *C.* *albicans*.

## Synopsis

*Candida albicans* is a fungal inhabitant of the intestinal tract of most healthy humans. It becomes a serious and often lethal pathogen in people with a weak immune system. *C. albicans* is a distant relative of the well-studied baker's yeast, *Saccharomyces cerevisiae.* It is now possible to determine the degree to which these two fungi have similar or different patterns of transcription.

Here, methods were developed that comprehensively compare the expression patterns of *S. cerevisiae* and *C. albicans.* A novel algorithm was used to determine if the expression of groups of genes in one organism are fully, partially, or not at all similar in the other organism. This algorithm was first applied to pre-defined groups of genes predicted to have similar functions and was then used to compare the global organization of the transcription programs between the two organisms.

The analysis revealed that the expression patterns reflect the different metabolic preferences of the two yeasts. The authors also found that amino acid metabolism regulation is more differentiated in *C.* *albicans*. Furthermore, the different expression patterns can be traced down to the use of different regulatory sequences. This study provides a comprehensive framework for comparative gene expression analysis, as well as a Web site with interactive analysis tools, which allow the development of hypotheses concerning uncharacterized genes and the sequences that regulate them.

## Introduction

Phenotypic diversity can often be traced to the differential expression of specific regulatory genes [1–5]. Recently, microarray experiments revealed large-scale differences in the genome-wide transcription response of related organisms to equivalent environmental conditions. For example, the transcription program underlying insect metamorphosis differs considerably between related species of the *Drosophila melanogaster* subgroup [6]. Similarly, both the meiotic and the mitotic cell cycle transcription program have diverged significantly between the budding and the fission yeasts [7]. The impact of such large-scale variations in gene expression on the phenotypes of the organisms is not yet understood.

Existing computational approaches for the comparative analysis of large-scale gene expression data have focused primarily on evolutionarily distant model organisms, for which large sets of expression data are available [8–11]. Such studies demonstrated that conservation of co-expression can improve functional gene annotation [9,10]. Common expression programs are invoked by related perturbations, such as adult onset in the nematode *Caenorhabditis elegans,* and the fruit fly *D. melanogaster* [11]. A generalization of the singular value decomposition approach that is applicable for such a comparative study was applied to cell cycle datasets from *Saccharomyces cerevisiae* and human [8]. Yet, the challenge of systematically comparing the gene expression program in related organisms is only starting to be addressed.

*Candida albicans* is an opportunistic pathogen that causes mucosal as well as systemic infections, especially in immune-compromised human hosts. This budding ascomycetous yeast diverged from the *S. cerevisiae* lineage between 140 and 800 million years ago [12,13]. Recently, the *C. albicans* genome was sequenced [14], revealing that almost two-thirds of its ~6,000 open reading frames are orthologous to *S. cerevisiae* genes. Microarray studies were performed by several groups characterizing the *C. albicans* genome-wide expression program under a range of conditions [15–24]. The availability of large sets of expression data in both *S. cerevisiae* and *C. albicans,* which are related organisms that span a significant evolutionary distance, provides a useful framework to develop and test computational tools for comparative gene expression analysis.

Here we present a novel approach for comparative gene expression analysis. We demonstrate the utility of our methods by systematically comparing the *C. albicans* and *S. cerevisiae* transcription programs at different levels of organization, ranging from the co-expression patterns between genes, to higher-order relationships between functional attributes. We describe large-scale differences in the transcription programs of the two organisms and use promoter analysis to link some of these differences to distinct *cis-*regulatory elements. All our results, as well as interactive analysis tools, are accessible through our Web server at http://barkai-serv.weizmann.ac.il/candida.

## Results

### *C. albicans* Expression Data

We assembled a dataset describing the genome-wide transcriptional responses of *C. albicans* to diverse perturbations, including drug resistance [15–17], stress [18], expression of only one mating type locus [19], and response to mating pheromone [20]. Also included were transcription profiles of cells growing as yeast or hyphal cells [25], in biofilms [21], exposed to blood components [22,23], altered pH [24], or signaling molecules [26,27]. The studies were performed primarily with laboratory strains, but also with some clinical isolates [15,21,24]. Altogether, the dataset consists of 244 expression profiles, generated by seven different laboratories, using four independently designed microarrays. All data were put into a unified format *(orf19),* which included a total of 6,167 open reading frames (ORFs) (see Materials and Methods).

**Figure 1:**
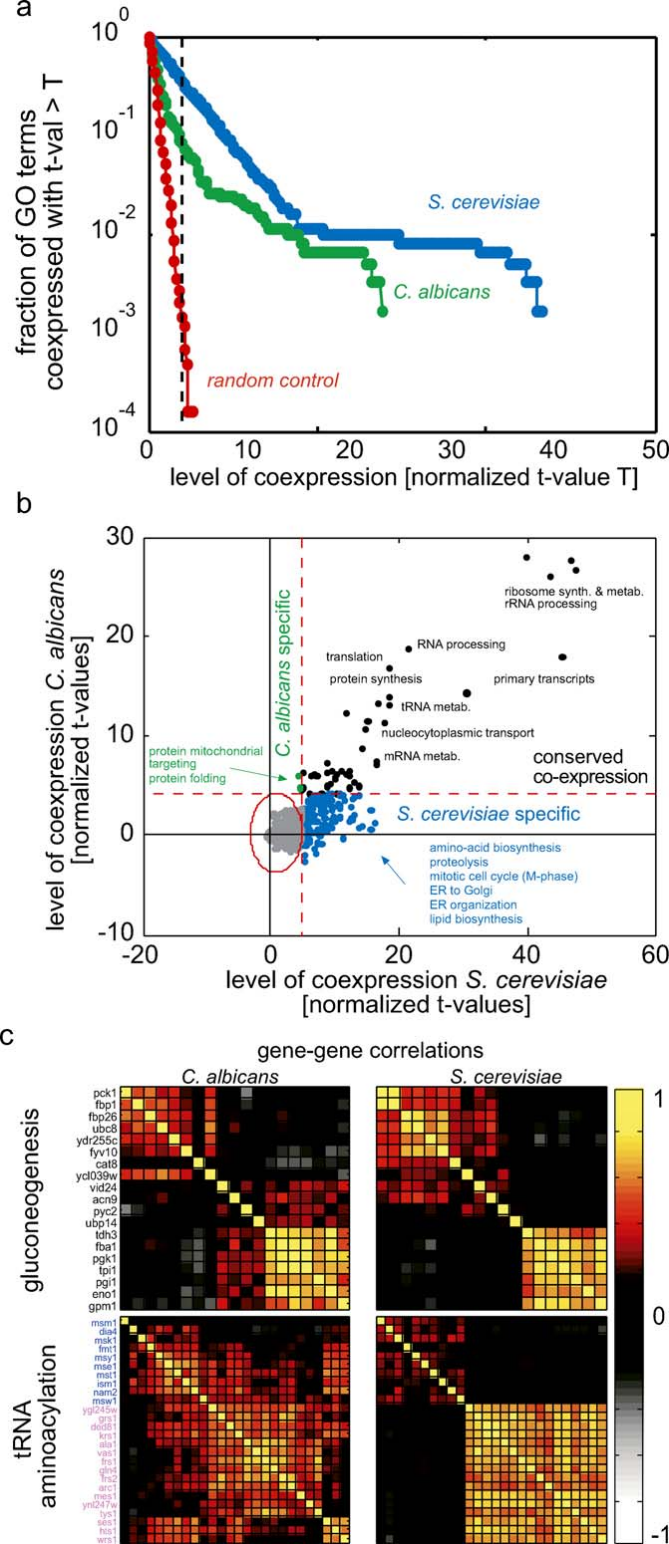
Functionally Linked Genes Tend to Be Co-Expressed (A) The extent of correlations between genes assigned to a particular GO category was quantified by the *t*-value associated with the distribution of pair-wise correlations between genes within the category (given in units of standard deviation (σ of the control distribution; see Materials and Methods). Shown is the fraction of GO categories whose *t*-value exceeds a threshold value T, as a function of T. As a control, we repeated the analysis for random assignment of genes into the GO categories (red). A similar analysis using genes assigned to a particular KEGG category is show in Figure S2. (B) The significance of GO term co-expression in *C. albicans* versus *S. cerevisiae.* Each dot corresponds to a specific GO category. GO terms that are significantly correlated in both organisms (*t*-value > 4σ) are colored in black, whereas those that are significantly correlated in only one organism are colored in blue or green. (C) PCMs of genes assigned to the indicated GO categories. Only genes defined as orthologous between *C. albicans* and *S. cerevisiae* were considered (Materials and Methods). Orthologs are arranged in the same order in both organisms. Mitochondrial and cytoplasmic genes are colored blue and magenta, respectively.

Previous studies demonstrated that genes with similar functions are often co-expressed (see [28–31]). To determine if this relationship is observed in the *C. albicans* expression data, we examined the similarity of the expression patterns of genes assigned to the same biological process within the Gene Ontology (GO) categories [32]. The significance of co-expression within a specific GO category was quantified by calculating the distribution of pair-wise correlations between genes within the category, and by comparing it to the distribution of random gene assemblies of the same size (see Materials and Methods and Figure S1). Indeed, a large fraction of predicted GO categories received a highly significant score, indicating that, also in the *C. albicans* data, functionally linked genes tend to be co-expressed (Figure 1A).

For comparison, we performed an analogous analysis of *S. cerevisiae,* using a dataset of ~1,000 publicly available genome-wide expression profiles [33]. Overall, the significance of co-expression within the *C. albicans* GO terms was lower than that of the *S. cerevisiae* counterparts (Figures 1A and S1). This lower significance may reflect the smaller size of the dataset available for *C. albicans,* its quality, or the fact that the GO terms were originally defined for *S. cerevisiae*. Alternatively, transcriptional regulation may play a less prominent role in *C. albicans*. The mitochondrial-targeting and protein-folding GO categories, which were co-expressed more tightly in *C. albicans,* provided an interesting exception, although the significance of this difference was marginal (Figure 1B). Despite the quantitative difference, we observed a strong correlation between the significance of the co-expression in the two organisms (correlation coefficient 0.92, Figure 1B). For example, in both organisms, functional groups involved in aspects of protein synthesis and sugar metabolism were most stringently co-expressed.

### Differential Clustering Algorithm for Comparative Analysis of Gene Expression Data

While providing a useful means for systematic analysis, GO categories do not necessarily correspond to transcriptional units. In fact, in most GO categories, only a subset of the genes is co-expressed (e.g., Figure 1C). Moreover, in certain cases, a single GO category can be separated into subsets that display independent or even inversely correlated expression patterns. For example, the *C. albicans* genes attributed to gluconeogenesis were split into two autonomously co-expressed subgroups, one associated with the glycolysis pathway itself, the other involved in other aspects of gluconeogenesis. Interestingly, in this case, this split was conserved between *S. cerevisiae* and *C. albicans* (Figure 1C). However, in general, the fine structures in regulatory patterns differed between the two organisms (e.g., tRNA aminoacetylation, Figure 1C).

**Figure 2:**
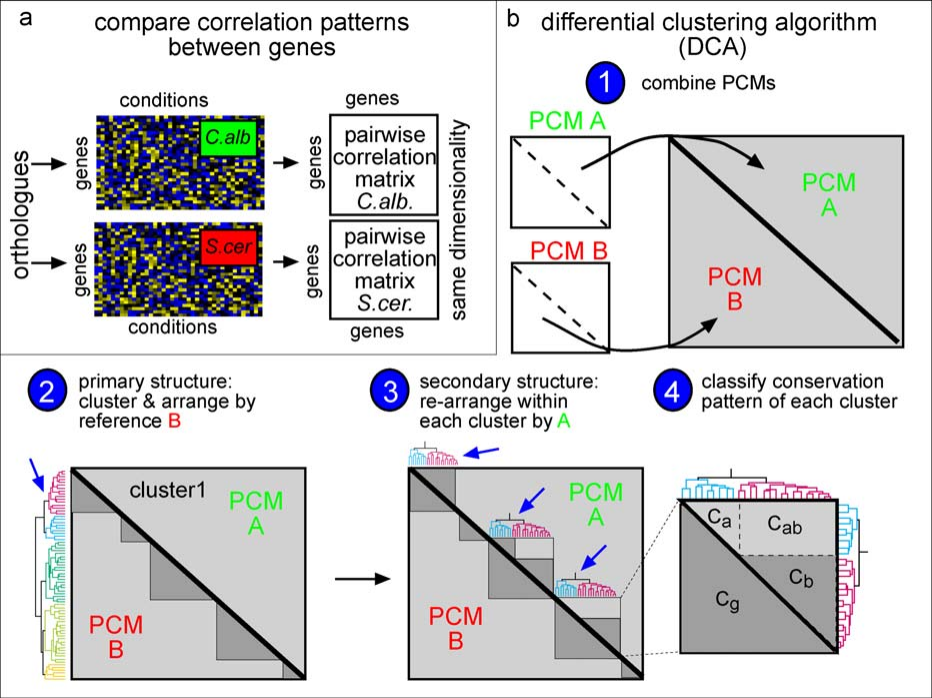
The Differential Clustering Algorithim (DCA) (A) PCMs are calculated (see Materials and Methods for details). (B) The PCMs are combined into a single matrix, where each triangle corresponds to one of the PCMs (1). The genes are then ordered in two steps: First, genes are clustered and the PCMs are re-arranged according to the correlations in the reference organism *(“B”)* (2). Second, the genes assigned to each of the resulting primary clusters are re-clustered according to their correlations in the “target” organism *“A”* (secondary clustering) (3). Note that, at each step of the clustering, orthologous genes are arranged in the same order in both organisms. The procedure is then repeated reciprocally, i.e., this time using organism *“A”* as the reference and organism *“B”* as the target. Finally, the conservation patterns of each cluster are classified automatically into one of the four conservation classes (4) (see also Figure 3A).

Differences in the pattern of gene regulation within individual GO categories are likely to reflect differences in the physiology, or in the adaptation to different environments, of the two organisms. Existing approaches for comparative gene expression analyses emphasize mostly conserved co-regulation patterns, rather than differences in expression patterns [8,9,11]. To better capture differential expression patterns, we developed a novel approach, termed the differential clustering algorithm (DCA), for systematically characterizing both similarities *and* differences in the fine structure of co-regulation patterns (Figure 2).

**Figure 3:**
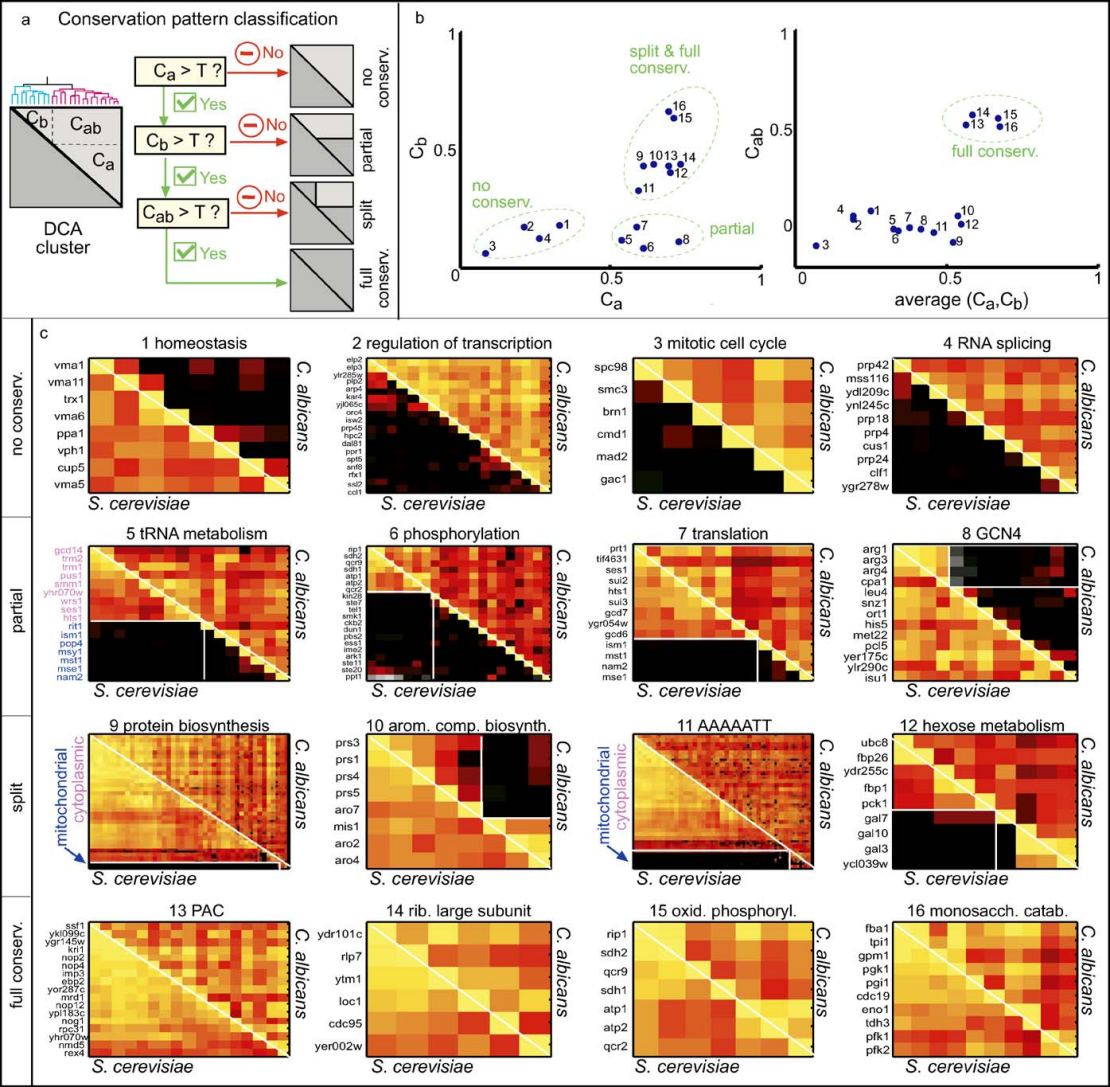
The DCA Method Automatically Classifies Clusters to Different Conservation Classes (A) Classification flowchart: Each primary cluster is subdivided into two secondary clusters, *a* and *b*. The cluster is then characterized by three correlation values, corresponding to the average correlations of genes within *(C_a_, C_b_* < *C_a_)* and between *(C_ab_)* these clusters. These correlations determine its assignment to one of four basic conservation patterns as depicted in the flowchart. The cutoff parameter T is chosen heuristically. (B) Classification values for clusters derived from functional GO categories. A list of clusters was obtained by applying the DCA method to sets of orthologous genes assigned to all functional GO categories containing between five and 200 orthologs. Sets of genes containing a specific sequence element in their 600-basepair promoter region were also considered (Materials and Methods). Shown are examples of clusters classified to each of the four basic types of conservation. Importantly, this assignment of clusters to the different conservation categories is robust to sub-sampling of the available conditions (Figure S3). (C) PCMs of the clusters shown in (B). Color code is as in Figure 1. Additional clusters related to these categories and gene names associated with all of the clusters are provided at http://barkai-serv.weizmann.ac.il/candida. Mitochondrial and cytoplasmic genes are colored blue and magenta, respectively. The category above each cluster refers to the GO term or sequence from which it was obtained. Note that each PCM represents only one cluster derived from the corresponding category, such that in general only a subset of the genes assigned to each category is shown.

The DCA is applied to a set of orthologous genes that are present in both organisms. As a first step, the pair-wise correlations between these genes are measured in each organism separately, defining two pair-wise correlation matrices (PCMs) of the same dimension (i.e., the number of orthologous genes) (Figure 2A). Next, the PCM of the primary (“reference”) organism is clustered, assigning genes into subsets that are co-expressed in this organism, but not necessarily in the second (“target”) organism. Finally, the genes within each co-expressed subgroup are re-ordered, by clustering according to the PCM of the target organism. This procedure is performed twice, reciprocally, such that each PCM is used once for the primary and once for secondary clustering, yielding two distinct orderings of the genes.

The results of the DCA are presented in terms of the rearranged PCMs. Since these matrices are symmetric and refer to the same set of orthologous genes, they can be combined into a single matrix without losing information. Specifically, we join the two PCMs into one composite matrix such that the lower-left triangle depicts the pair-wise correlations in the reference organism, while the upper-right triangle depicts the correlations in the target organism (Figure 2B). Inspection of the rearranged composite PCM allows for an intuitive extraction of the differences and similarities in the co-expression pattern of the two organisms (Figure 3). An automatic scoring method is then applied to classify clusters into one of the four conservation categories: *full, partial, split,* or *no conservation* of co-expression (Figure 3A and 3B).

### Functionally Related Genes Exhibit Different Degrees of Co-Expression Conservation

To systematically characterize the conservation or divergence of co-expression between genes with a related function, we applied the DCA to gene groups defined by membership in the same biological process GO categories [32]. We also applied it to groups of genes that have a common DNA sequence motif of length 6 or 7 base-pairs in their promoter region (within 600 base-pairs upstream of the predicted start codon). The DCA procedure identifies co-expressed clusters embedded within these gene sets, and assigns each of these clusters to one of the four above-mentioned conservation categories (*full, partial, split,* or *no conservation*, Figure 3)*.*

Examples of clusters assigned to each category are shown in Figure 3C. Clusters associated with growth, including genes encoding ribosomal components (Figure 3C, 14) and genes containing the PAC motif (Figure 3C, 13, primarily genes encoding rRNA processing proteins), were strongly co-regulated in both organisms, and were thus assigned to the *full conservation* class. Also assigned to this class were clusters of genes involved in oxidative phosphorylation (Figure 3C, 15) and monosaccharide catabolism (Figure 3C, 16).

Of particular interest are clusters that are differentially expressed between the two organisms. The most noticeable differences were found for clusters whose genes are involved in both cytoplasmic and mitochondrial translation. This included, for example, the GO terms “protein synthesis” (Figure 3C, 9), “tRNA metabolism” (Figure 3C, 5), and “tRNA amino-acetylation” (Figure 1C). These clusters were uniformly co-expressed in *C. albicans.* In contrast, in *S. cerevisiae* they were split into two distinct subclusters, associated with cytoplasmic or mitochondrial functions, respectively, which displayed independent or even inversely correlated expression patterns. This differential expression pattern of mitochondrial genes reflects a major phenotypic difference between the two organisms: rapidly growing *S. cerevisiae* cells utilize fermentation and do not require oxygen. In contrast, rapid growth in *C. albicans* relies on aerobic respiration and requires mitochondrial functions.

### Flexible Regulatory Patterns of Cell Cycle Genes

Among the clusters assigned to the *no conservation* class was a group of cell cycle genes that are involved in the transition from S-phase to mitosis (Figure 3C, 3). These genes were tightly co-expressed in *C. albicans,* but not in *S. cerevisiae,* suggesting that the cell cycle transcription program differs between the two organisms.

To better characterize the differences in regulation of cell cycle genes, we examined the “cell cycle” GO category in more detail. We included in this analysis also expression data from *Schizosaccharomyces pombe* [7,34]*,* which is evolutionarily more distant to *S. cerevisiae* and *C. albicans* [13]. For *S. cerevisiae* and *S. pombe,* we also restricted the expression data to cell cycle experiments. No such cell cycle–dedicated conditions were available for *C. albicans*. We note, however, that many experiments in the *C. albicans* dataset used cells emerging from stationary phase with some degree of synchrony, which likely captured some features of cell cycle–specific regulation. It should be noted that the gene set is based on the *S. cerevisiae* GO term, and therefore does not include genes that are cell cycle–related only in the other two organisms.

**Figure 4:**
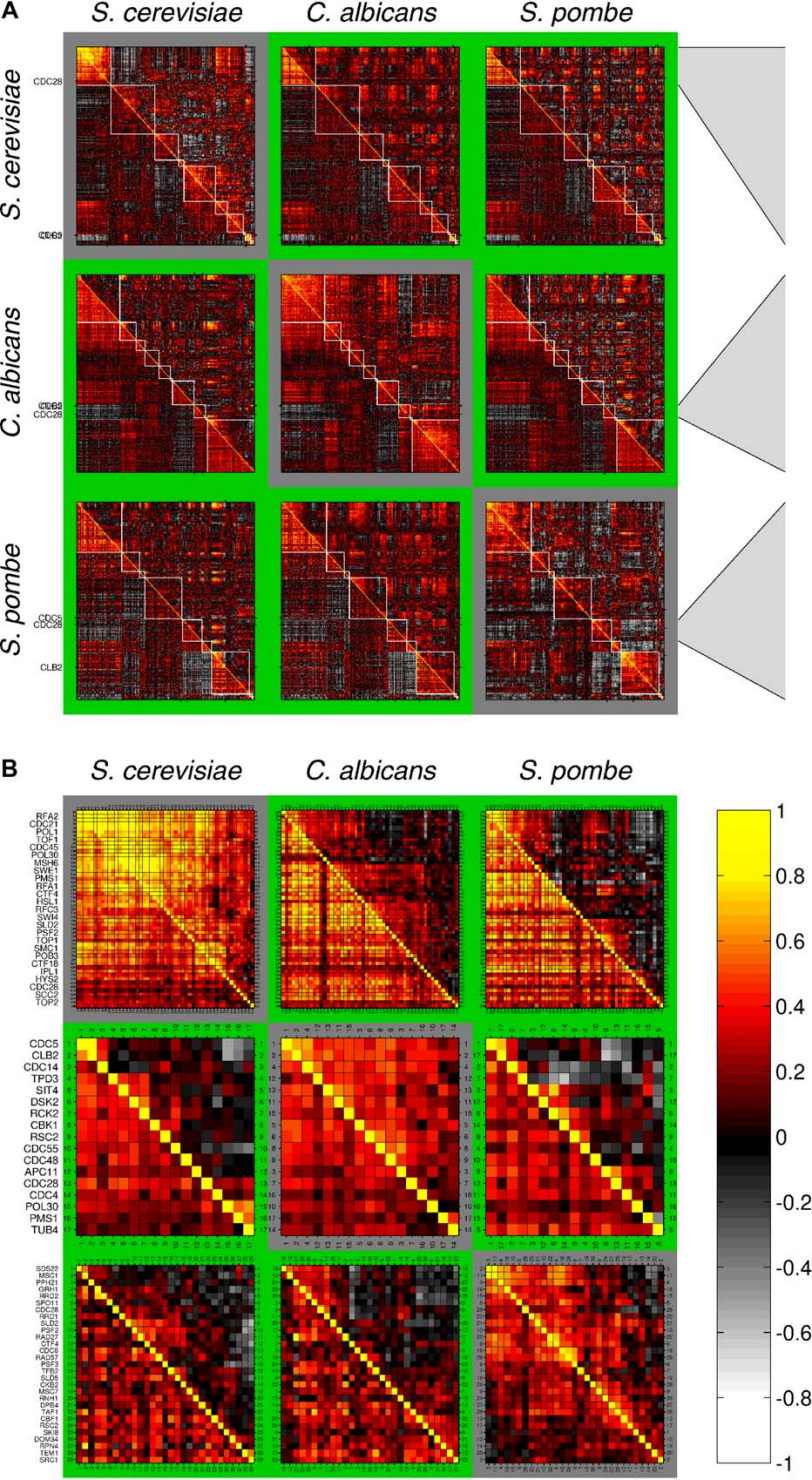
DCA Analysis of Cell Cycle Genes (A) The DCA algorithm was applied to a restricted gene set, consisting of all genes common to *S. cerevisiae, C. albicans,* and *S. pombe,* with GO annotation “cell cycle.” The reference organism is indicated on the left, whereas the target organism is indicated on the top. Most of the primary clusters (white boxes) are, at most, partially co-expressed in the target organism (green background). The diagonal (gray background) represents the control, where the secondary clustering is performed for the same species as in the primary clustering, but using a reduced set (25%) of the expression data. (B) Primary clusters from (A) that contain CDC28. Note that CLB2 and CDC5 are tightly co-expressed in *S. cerevisiae* and *C. albicans* (but not in *S. pombe*), but that CDC28 is co-expressed with these genes only in *C. albicans*. Details of all other clusters are available in Figures S4–S13.

We applied the DCA to the above-mentioned data, with each of the three yeasts serving once as a reference and once as a target organism (off-diagonal in Figure 4, green background). As a control, we considered the same organism as both the reference and target organism, but used only 25% of the expression data for the secondary clustering (diagonal in Figure 4, gray background). Moreover, for *S. cerevisiae* and *S. pombe,* we tested complementary expression data containing no cell cycle experiments as another control. In this case the cluster conservation was weaker, yet some aspects of cell cycle regulation remained (unpublished data).

Essentially all clusters identified as co-expressed in the reference organism were, at most, partially co-expressed in the other two organisms (Figures 4 and S4–S13). As an example, we highlight here the regulation of the major cyclin-dependent kinase (encoded by *CDC28* in *S. cerevisiae*) and the associated mitotic B-cyclin (encoded by *CLB2*) (Figure 4B).

In *S. cerevisiae*, there are six B-cyclins, several with redundant functions [35–38], and their expression is cell cycle–regulated. *CDC28* expression is not correlated with any of them. Accordingly, *CDC28* and *CLB2* were associated with two distinct clusters: *CDC28* was assigned to a cluster composed of genes involved in the early cell cycle functions (e.g., budneck formation, DNA replication, and repair [Figure S4]), whereas *CLB2* was assigned to a cluster composed of genes with functions in mitosis (Figure S12). Neither of these clusters was co-expressed in *C. albicans* or in *S. pombe*.

*S. pombe* has one major, essential B-cyclin, *cdc13* (the *CLB2* ortholog), which is required for mitosis. In the *S. pombe* cell cycle data*,* expression of *cdc13* was inversely correlated with expression of *cdc2* (the *CDC28* ortholog). *cdc2* was co-expressed with a cluster of genes, many of whose *S. cerevisiae* orthologs are involved in replication and DNA repair (Figure S9), whereas *cdc13* was co-regulated with genes involved primarily in mitosis and general cell cycle control (Figure S11).

*C. albicans* has two B-cyclins, and one of them, *CLB2,* is essential [39]. Interestingly, in *C. albicans* the *CDC28* and *CLB2* orthologs were co-expressed. Both genes were assigned to a cluster associated with anaphase and mitotic exit (Figures 4B and S11). Northern blot analysis of *CDC28* and *CLB2* transcripts in *C. albicans* cells emerging synchronously from stationary phase confirmed that the mRNA levels of *CDC28* and *CLB2* correlate, peaking with the presence of large budded cells (S/G2 phase) (JB and M. McClellan, unpublished data).

We conclude that transcriptional regulation of cell cycle genes is highly flexible and has diverged significantly between the three yeast species. Our results expand on previous reports that have shown that only a small set of genes are subject to similar cell cycle regulation in both *S. cerevisiae* and *S. pombe* [7,40]*.* Each of these fungi has a distinctive repertoire of morphologies: *S. cerevisiae* and *C. albicans* undergo budding to form yeast or pseudohyphae; *C. albicans* also forms true hyphae by a non-budding mechanism involving different organellar structures [41]; *S. pombe* is a fission yeast with a distinct, non-budding mechanism of morphogenesis. In all three fungi, cell cycle regulation and morphogenesis are clearly linked [39,42,43]. Further analysis is needed to establish how these distinct morphologies are connected to the differential pattern of gene expression found in each organism.

### *C. albicans* Transcription Modularity

The analysis above focused on pre-defined sets of genes that are known to be related and thus are suspected to be, at least partially, co-expressed. In order to identify novel regulatory relationships that are not confined to specific function-related genes, we conducted a complementary, unsupervised analysis of the *C. albicans* expression data. To this end, we used the iterative signature algorithm (ISA) [31,44] to determine the modular organization of the *C. albicans* transcription program. The ISA segregates the data into overlapping transcription modules, each consisting of a subset of co-expressed genes together with the subset of experimental conditions inducing this co-expression.

**Figure 5:**
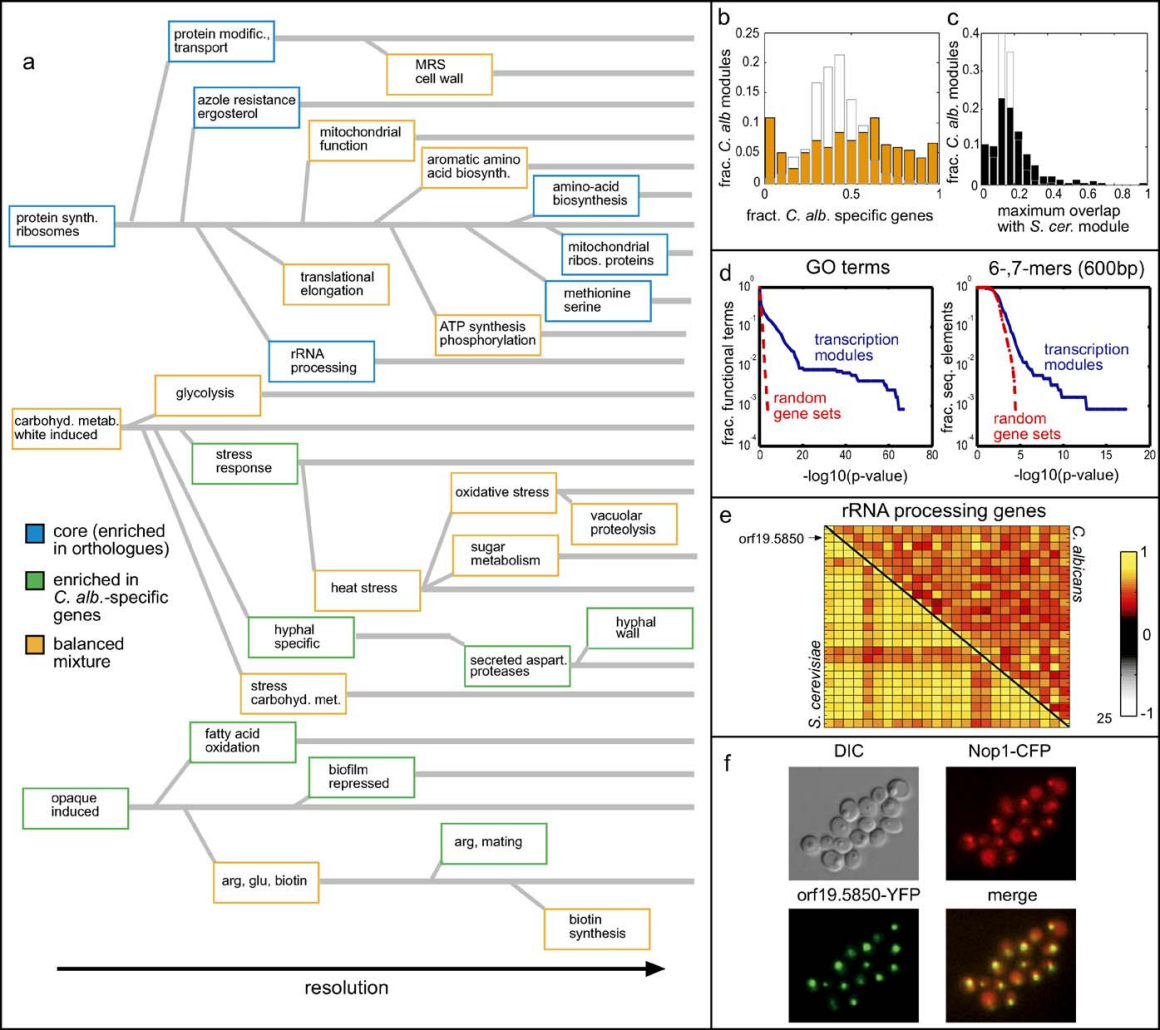
*C. albicans* Module Tree (A) Transcription modules were identified using the ISA [31,44]. Modules were annotated manually, and are colored according to their enrichment for *S. cerevisiae* orthologs or *C. albicans–*specific genes. An interactive version of the tree with details of the genes and conditions of each module is provided at http://barkai-serv.weizmann.ac.il/candida. (B) Proportion of genes without *S. cerevisiae* orthologs in *C. albicans* transcription modules (orange), compared to a control distribution obtained from random sets of genes of the same size. Note the over-representation of *C. albicans*–specific modules. (C) Distribution of overlaps between transcription modules of *C. albicans* and *S. cerevisiae*. For each representative module in *C. albicans,* the *S. cerevisiae* module with the highest overlap in terms of orthologous genes was identified and the proportion of overlap was plotted (Materials and Methods). (D) Transcription modules are significantly enriched in common GO terms and upstream sequence elements. For each transcription module in *C. albicans,* enrichment *p*-values were calculated for all GO terms or sequence elements (6-, 7-mers) in the 5′ UTR (Materials and Methods), and the *n* smallest *p*-values were recorded for each module. The results are shown for *n* = 5, but are robust to the precise choice of *n*. The fraction of categories and sequence elements exceeding a threshold *p*-value, as a function of the threshold, is shown and compared to a control distribution obtained from random gene sets of the same sizes. (E) PCMs of genes involved in rRNA processing. Shown are the gene–gene correlation matrices of the top-scoring 25 genes assigned to the *rRNA processing* module in *C. albicans* (left panel) and their *S. cerevisiae* orthologs (right panel). Genes are ordered by their gene score in the *C. albicans* transcription module. (F) orf19.5850-YFP, assigned to the rRNA processing module, co-localizes with Nop1-CFP to the nucleolus.

The ISA assigned 2,770 *C. albicans* genes into transcription modules with varying stringencies of correlated expression. Modules were classified as *core* modules (15%), composed primarily of genes possessing an *S. cerevisiae* ortholog; as *C. albicans*–specific modules (37%), consisting primarily of genes without *S. cerevisiae* orthologs; or as modules with a mixture of both types of genes (48%) (Figure 5A–5C).

Modules were annotated manually by examining their gene and condition contents (Figure 5A; see also http://barkai-serv.weizmann.ac.il/candida). In addition, we systematically checked each module for over-representation of GO categories and of DNA sequence motifs in the 5′-UTR. This analysis clearly established the biological relevance of the *C. albicans* transcription modules. First, many modules contained one or several over-represented GO terms, indicating their functional coherence (Figure 5D). Second, most modules were associated with sequence motifs that were significantly enriched in the promoter regions of genes within the module (Figure 5D).

Module association provides numerous functional links for *C. albicans* genes (see http://barkai-serv.weizmann.ac.il/candida). We experimentally tested one of these links, namely *orf19.5850.* Previous studies reported that a strain heterozygous for a transposon disruption allele of this gene exhibits reduced filamentous growth [45]. Our analysis assigned *orf19.5850* to the rRNA processing module (Figure 5E). Indeed, tagging this predicted protein product with yellow fluorescent protein (YFP) revealed its localization to the nucleolus (Figure 5F), as expected for a gene involved in rRNA processing. After this experiment was initiated, the localization of the *S. cerevisiae* ortholog was shown to be both nucleolar and nuclear [46].

### The *C. albicans* versus *S. cerevisiae* Transcription Modularity

The hierarchical organization of a transcription program is captured by its *module tree,* which connects related modules identified at different stringencies of correlated expression [10,31,44] (Figure 5A). The *C. albicans* module tree was composed of three main branches. One of these branches was associated with *Candida*-specific cell types: they were induced in opaque cells and/or repressed in white cells. This module included genes important for fatty acid metabolism, mating, and arginine and glutamine biosynthesis, as well as genes repressed under conditions of biofilm production. The second main branch was composed primarily of modules pertaining to core functions, including genes required for rapid growth (e.g., ribosomal proteins and rRNA processing genes). Finally, the third main branch was associated with carbohydrate metabolism and the response to stress, as well as with genes involved in *C. albicans–*specific processes such as hyphal or white-opaque growth.

This global organization is similar to that found in the *S. cerevisiae* module tree, in which two of the major branches were associated with rapid growth and stress-response, respectively [31,44]. In contrast, in higher eukaryotes, including *D. melanogaster, C. elegans, Arabidopsis thaliana,* and human, these two core functions did not correspond to main branches of the module trees [10].

Apart from this global similarity, the module trees of *C. albicans* and *S. cerevisiae* displayed some notable differences. First, in *C. albicans*, amino acid biosynthesis was associated with the protein synthesis branch, whereas no such association was seen in *S. cerevisiae* [31,33,44]. This indicates that in *C. albicans,* but not in *S. cerevisiae,* amino acid biosynthesis is induced under conditions that also increase protein synthesis (e.g., rapid growth). To test if these differences arose from the distinct types of conditions available in the two datasets, we removed from the *S. cerevisiae* data all environmental perturbations relevant for amino acid metabolism (such as amino acid or nitrogen starvation). We also removed other subsets of conditions, such as the set of 300 profiles of deletion mutants [47], or the set of general environmental perturbations [48]. In all cases, the amino acid and the protein synthesis modules appeared on separate branches (unpublished data). This indicates that the observed distinctions in the module trees of the two yeasts reflect differences in the underlying organization of their transcriptional programs, rather than differences in the set of available conditions.

In *C. albicans,* the core protein synthesis branch also included specific modules, which contained members of the major repeat sequence family [49] along with genes important for cell wall synthesis and several genes involved in cell cycle progression, such as *CLB2, CDC5,* and *CDC28*. The reason for this association of cell wall proteins, the major repeat sequence family, and cell cycle genes is not clear. Examining the conditions associated with this module, we noted that this module is induced primarily in white cells and is repressed primarily in opaque cells [19], and thus may reflect a common regulation associated with the conditions used to study the white-opaque transition.

An intriguing feature of the *C. albicans*–specific branch of the transcription program is that genes related to arginine biosynthesis were separated from the main amino acid biosynthesis module. These genes were co-expressed with genes required for biotin synthesis, most likely because biotin is required for the activity of ornithine transcarbamylase (encoded by *ARG3*) [50]. In addition, these genes were co-expressed with genes associated with the mating response [19,20] and were up-regulated in *C. albicans* cells interacting with macrophages [23]. Because methylated arginines are inhibitors of nitric oxide [51], which is produced by macrophages, it is tempting to speculate that the expression of genes required for arginine synthesis elicits a protective response of *C. albicans* cells to macrophage attack.

Furthermore, in *C. albicans,* the mitochondrial ribosomal protein module and the ergosterol biosynthesis module both appear on the protein synthesis branch associated with rapid growth. In contrast, the *S. cerevisiae* mitochondrial ribosomal protein module is associated with stress responses. Again, this pattern of co-regulation likely reflects the fact that rapid growth requires mitochondria-mediated respiration in *C. albicans* but not in *S. cerevisiae*.

### Higher-Order Regulatory Relationships between GO Terms Provide Complementary Views of Transcription Programs

The above direct comparison of the two module trees is useful for distinguishing broad features of the respective organizations, yet it is limited by the lack of a one-to-one relationship between the two module sets. For example, the average overlap between *S. cerevisiae* modules and their best matching *C. albicans* counterparts is only 19% (Figure 5C). Furthermore, although many modules are significantly enriched with genes belonging to a specific GO category, typically several distinct GO categories are represented in each module. Thus, associating each module with one summarizing annotation is a simplification that does not capture the full complexity of the transcriptional organization.

**Figure 6:**
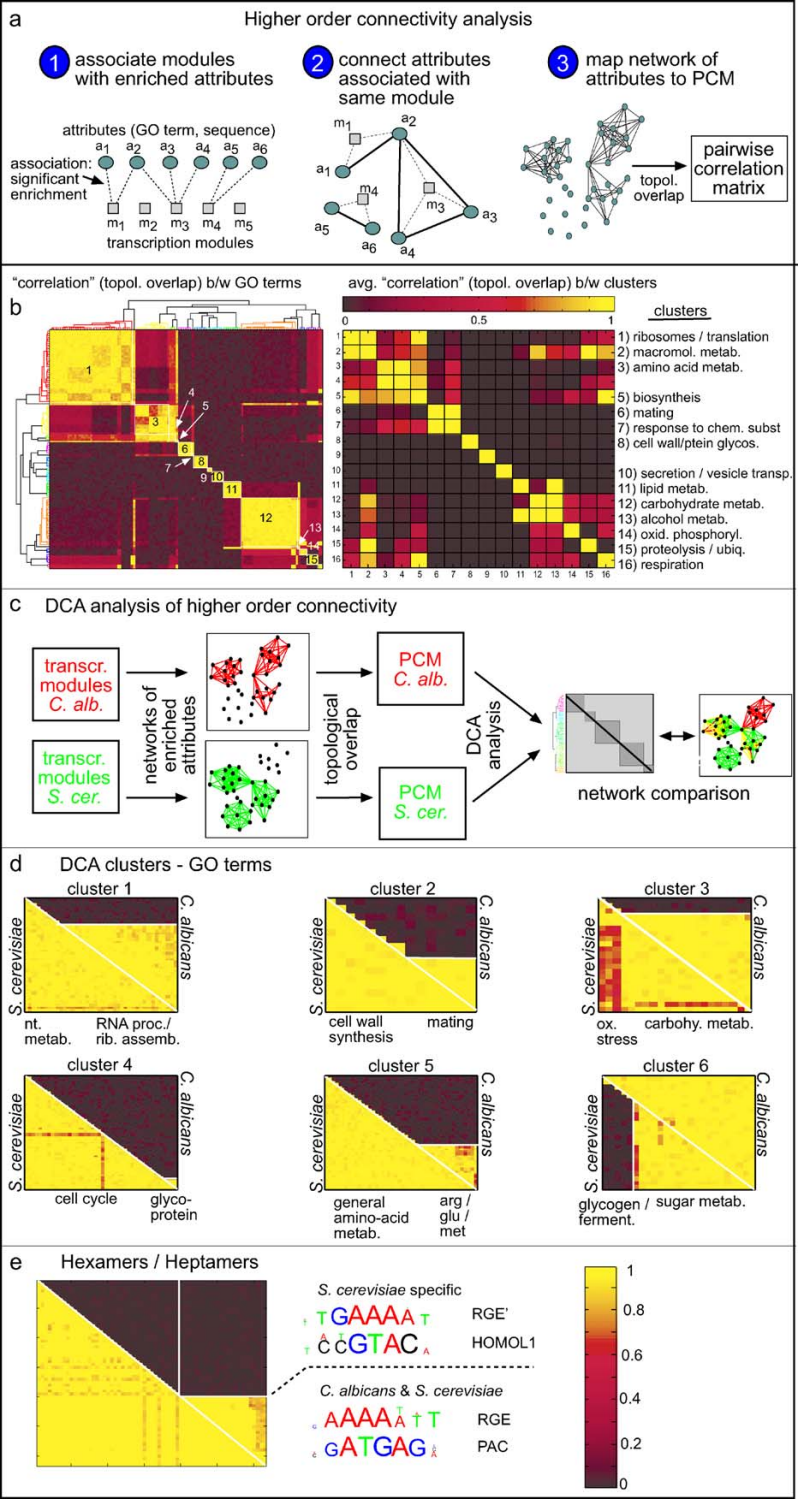
Connectivity Analysis between Gene Attributes Reveals Different Patterns of Co-Expression in *C. albicans* and *S. cerevisiae* (A) Generalized attributes (GO terms, sequence motifs, etc.) are connected if they are significantly over-represented in the same transcription module. To analyze the resulting enrichment networks, we first define correlations between attributes based on the topological overlap measure ([52]; see Materials and Methods). (B) Clustering of the PCM of hierarchical overlaps in *C. albicans*. Shown is the clustered PCM (left) and a matrix of average correlation/topological overlap values for each cluster (right). (C) To compare networks between organisms, the DCA method was applied to PCMs of topological overlaps. (D) Shown are examples of clusters obtained from DCA analysis of the GO networks of *C. albicans* and *S. cerevisiae*. (E) Same as in (D), but using the occurrence of hexa- and heptamer binding motifs in the promoter as gene attributes. (Interactive figures with the list of the GO terms or binding motifs assigned to each cluster, are provided at http://barkai-serv.weizmann.ac.il/candida.)DOI: 10.1371/journal.pgen.0010039.g006

To overcome these difficulties, we developed a new approach, termed “higher-order connectivity analysis” (HOCA), in which we analyze the modular components of the transcription program through their association with functional categories. Specifically, we define a GO connectivity network, where two GO terms are connected if they are both over-represented in at least one common transcription module (Figure 6A, and Materials and Methods). Applying HOCA to the *S. cerevisiae* and *C. albicans* expression data yielded two independent “GO networks,” corresponding to the regulatory relationships between the GO terms in *S. cerevisiae* and *C. albicans,* respectively. The two networks were composed of a corresponding set of nodes (GO terms), connected by organism-specific links. We quantified the strength of each link using the *topological overlap* [52], which weights each edge by the similarity in the overall connectivity of the two nodes (Figure 6A, and Materials and Methods). The *C. albicans* GO connectivity diagram is displayed in Figure 6B.

### Differential Connectivity in the *C. albicans* versus *S. cerevisiae* GO Networks

To compare the GO networks of *C. albicans* and *S. cerevisiae,* we restricted the set of nodes to the GO terms that are common to both organisms. In this case, we have two matrices of the same dimension (i.e., the number of common GO terms), describing the topological overlaps between all pairs of GO terms in each organism (Figure 6C). The two matrices were analyzed using the DCA method to automatically classify the resulting clusters of GO terms into the *full, split, partial,* and *no conservation* classes of co-expression.

Figure 6D depicts some of the GO term associations assigned to the different conservation classes. Notably, GO terms concerning carbohydrate metabolism (c.f. cluster 3) were correlated with the stress response in *C. albicans* but not in *S. cerevisiae*. This may be related to the fact that *C. albicans* requires mitochondrial function during rapid (aerobic) growth, producing high levels of reactive oxygen species that, in turn, would induce oxidative stress–related genes. In contrast, rapid (fermentive) growth in *S. cerevisiae* does not generate such high levels of reactive oxygen species and therefore would not induce these genes.

### Sequence Motifs Associated with the Differential Regulation of *C. albicans* Amino Acid Biosynthesis Genes

Consistent with the modular analysis described above, we detected an interesting difference in the regulation of amino acid biosynthesis genes in *C. albicans* relative to *S. cerevisiae*. Cluster 5 (Figure 6D) includes GO terms involved in the biosynthesis of several amino acids. All these GO terms are connected in *S. cerevisiae,* presumably reflecting their common regulation by the transcription factor Gcn4p. In contrast, only one subset of these GO terms (arginine, glutamine, and sulfur amino acid metabolism) was connected in *C. albicans*. This suggests a differential, and more refined regulation of amino acid biosynthesis by *C. albicans.*

**Figure 7:**
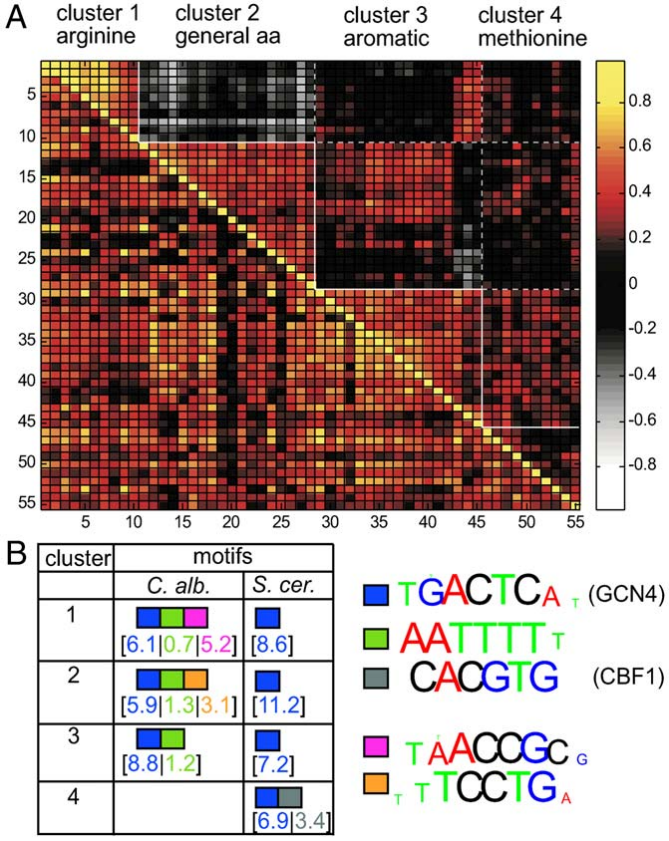
DCA Analysis of Amino Acid Biosynthesis Genes (A) Gene–gene correlation matrix for genes assigned to the *S. cerevisiae* amino acid biosynthesis module*.* Lower triangle corresponds to the *S. cerevisiae* data, while the upper triangle depicts the *C. albicans* correlations. (B) Sequences motifs over-represented in the different DCA clusters.

To better characterize this differential co-regulation pattern, we applied the DCA to the genes of the amino acid biosynthesis transcription module in *S. cerevisiae* (Materials and Methods). In *S. cerevisiae,* these genes are uniformly co-expressed. In contrast, in *C. albicans* this group was split into four clusters that displayed distinct regulatory patterns (Figure 7). These clusters were associated with arginine, methionine, aromatic, and general amino acid biosynthesis.

To address the mechanism underlying this differential regulatory pattern, we asked whether these clusters are linked to differential appearance of *cis-*regulatory elements. To this end, we examined the promoter sequences of the genes in each cluster, searching for an over-represented DNA sequence of length 6–8 nucleotides. First, we analyzed the *S. cerevisiae* promoters and found that, as expected, all clusters were significantly enriched with the TGACTC motif, which is the known binding site for Gcn4p, the transcriptional activator of amino acid biosynthetic genes. Furthermore, the cluster that includes genes required for methionine biosynthesis was associated with an additional motif (CACGTG), which is bound by the Cbf1 transcription factor, a known regulator of methionine biosynthesis genes [53].

Next, we searched for over-represented DNA sequences in the promoters of genes in the *C. albicans* clusters. The TGACTC motif was significantly enriched in three of the four clusters (numbers 1–3), consistent with previous reports showing its conservation across different yeast species [54,55]. Notably, the cluster associated with methionine biosynthesis genes, which is not co-regulated in our dataset, appears to have lost both the TGACTC (Gcn4-binding) and the CACGTG (Cbf1-binding) motifs (Figure 7).

Strikingly, the three *C. albicans* clusters that maintained the TGACTC motif were all associated with additional over-represented motifs that were not found in the promoters of the corresponding *S. cerevisiae* genes (Figure 7). Specifically, the arginine and general amino acid clusters were each associated with a distinct novel motif (TAACCGC and TTCCTG, respectively), whereas all three clusters were associated with the AATTTT [56] motif**.** These results suggest that combinatorial regulation by different transcription factors underlies the distinct pattern of amino acid biosynthesis genes in *C. albicans.* Interestingly, the AATTTT motif (or its reverse complement; see Figure 3C, 11) is also enriched in genes involved in ribosome biogenesis and rRNA processing, providing a possible explanation for the observed correlation between amino acid biosynthesis and the protein synthesis branch in the *C. albicans* module tree.

### Differential Connectivity between *Cis*-Regulatory Elements

The above analysis described the higher-order organization of the *C. albicans* transcription program based on gene sets sharing functional attributes (i.e., GO categories). A complementary approach is to define putative regulatory units based on common sequence motifs in the 5′-UTRs of its genes.

In a given transcription module, more than one sequence element is typically over-represented. Multiple associations of binding motifs that differ by a single nucleotide likely reflect flexibility in the binding specificity of a single transcription factor. These sequences can be summarized by a consensus motif. Indeed, several clusters of motifs assigned to the “split” conservation pattern correspond to consensus motifs that are partially conserved, but exhibit some organism-specific modifications. Interestingly, many single nucleotide sequence variations of a motif were connected only in *S. cerevisiae,* suggesting that *S. cerevisiae* transcription factors tend to have a higher degree of DNA binding flexibility as compared to their *C. albicans* counterparts. Moreover, the consensus sequences in *S. cerevisiae* were usually slightly different from those in *C. albicans*.

Over-representation of several distinct sequence motifs in a given transcription module most likely indicates combinatorial regulation of these genes by different transcription factors. For example, in both organisms, the known consensus motifs PAC [57] and the sequence AAAATT were linked in a single cluster (Figure 6E) pointing to combinatorial action of the associated transcription factors. Moreover, the sequence TGAAAAT was connected to this cluster, but only in *S. cerevisiae.* This indicates that in *S. cerevisiae,* the common sequence AAAAAT almost always appears with the prefix TG. In contrast, this TG prefix is not seen in *C. albicans*. Additional results are summarized at http://barkai-serv.weizmann.ac.il/candida.

## Discussion

We present a novel computational approach for the comparative analysis of large-scale gene expression data. Expression data in two organisms were compared at three different levels. First, the DCA was used to analyze co-regulation within specific groups of genes. These groups were assembled based on a priori biological knowledge and are likely to include a subset of co-regulated genes. Focusing on specific functional groups of interest allows the direct analysis of co-expression patterns without interference from genes of unrelated function. Second, the ISA [31,44] was used to identify modules of co-regulated genes. Modular decomposition was performed independently for the two organisms, leading to two module trees that can be compared directly. This unsupervised analysis enables the identification of novel regulatory relationships, which may not be captured by our first analysis based on a priori functional classification. Third, the HOCA was used to rigorously compare the connectivity between different functional units. This analysis relies on the segregation of the expression data into condition-specific transcription modules. Importantly, the HOCA approach can be applied to characterize and compare the connectivity between different types of functional attributes, such as GO terms or *cis-*regulatory motifs.

A common approach for comparative analysis of gene expression is to consider the transcriptional responses to sets of perturbations that are assumed to be equivalent in both organisms. Yet, robust analysis of gene expression data requires a large number of profiles, and restricting the data to a subset of experiments with common conditions severely restricts the number of available profiles. Moreover, obtaining precisely the same experimental conditions is difficult, if not impossible, when analyzing public datasets. In particular, even when equivalent conditions can be identified, different responses in gene expression could reflect differences in signal transduction mechanisms rather than in the underlying transcriptional network.

The present approach circumvents the need for equivalent experiments because it compares the patterns of gene–gene correlation between the two organisms. The input to the DCA consists of two matrices of the same dimensions, describing the pair-wise similarities between orthologous genes, or groups of genes, measured separately in each dataset. The DCA approach performs clustering sequentially and reciprocally, each time using one set of expression data for primary partitioning and the other dataset to identify the secondary patterns of co-expression within these partitions. Thus, the DCA allows for the identification of diverged, partially conserved, and well-conserved patterns of co-expression between the two datasets. Compared to previous studies that focused primarily on conserved co-regulation [9], this provides an important advantage, especially when more closely related species are analyzed.

It is important to note that, in a heterogeneous compendium of expression profiles, condition-specific co-expression can be obscured when using a simple correlation metric over all conditions. In our initial application of the DCA to the PCMs of pre-defined gene sets, we neglected this issue for simplicity, although this limitation could, in principal, be alleviated using different distance matrices (such as “mutual information” [58]). However, condition-specific co-regulation is taken into account in our global modular analysis using the ISA, as well in our HOCA approach, which is based on module association.

To illustrate the utility of our approaches, we applied them systematically to compare the transcription program of *C. albicans* with the well-characterized *S. cerevisiae* program*.* While the co-expression of many functionally related groups was conserved between *C. albicans* and *S. cerevisiae,* our analysis also revealed major distinctions between the two transcription programs. For some of these differences, such as the distinct regulation of genes involved in mitochondrial versus cytoplasmic protein synthesis, the association with distinct phenotypes (e.g., aerobic versus anaerobic rapid growth) is apparent. Other differences, such as those related to cell cycle or amino acid biosynthesis, remain to be elucidated. The former may be connected to different mechanisms of cell cycle regulation pertaining to morphology and/or to different points of cell cycle control exhibited by the two organisms. The latter may reflect the fact that *C. albicans* lives primarily within a human host, and thus may grow in an environment that readily provides specific subsets of amino acids.

It should be noted that although the number of *C. albicans* transcription profiles used in this analysis (~250 different arrays) is large, this dataset is probably far from being saturated. Additional differences are likely to be revealed once more data become available. Our comprehensive account of co-regulation in *C. albicans* provides numerous functional links, as well as important regulatory information, about individual *C. albicans* ORFs. All the results are available in an interactive format on our Web page at http://barkai-serv.weizmann.ac.il/candida.

Understanding the principles underlying the evolution of gene expression requires systematic comparison of expression data between related organisms. The methods presented in this paper will assist in this challenge. Furthermore, our approach is not limited to the analysis of two sets of expression data, but can be adapted to compare large-scale data of different types, e.g., expression data with protein–protein interaction data or with phenotypic data.

## Materials and Methods

### Expression data.

Individual experimental datasets were all put into a standardized *orf19* gene name format using conversion information provided by A. Nantel, C. D'Enfert, and A. Tsong. Expression data were stored as log2 ratios. Initial analysis identified a significant number of modules that reflected genes with a strong bias for Cy3 versus Cy5 dye labeling. To minimize this effect, dye swap data for the same experimental conditions were averaged whenever possible, resulting in a total of 244 conditions.

### Definition of orthologous genes.

We used the Inparanoid software to determine orthologous pairs of genes [59]. Sequence information for *C. albicans* was based on the *orf19* assembly. In the case of multiple genes in a cluster (~5%), we used the one with the highest score, resulting in 3,619 one-to-one ortholog pairs.

### Definition of gene sets.

Functional GO categories were downloaded from http://www.geneontology.org. The assignment of genes to the original GO categories was extended to include parent terms, i.e., a gene assigned to a given category was automatically assigned to all the parent categories as well. Only genes classified as orthologous between *C. albicans* and *S. cerevisiae* were considered, and *C. albicans* categorization was inferred from *S. cerevisiae* orthologs. All GO terms containing at least five orthologs were considered. In the HOCA of GO terms in *C. albicans* (Figure 6B), this categorization was supplemented with *C. albicans*–specific GO annotations obtained from the Candida Genome Database (http://www.candidagenome.org). For the analysis shown in Figure 3, we also added gene sets based on promoter sequence elements. For each sequence element (of length 6 and 7), the genes containing the element in their 600-basepair upstream regions were identified for both *S. cerevisiae* and *C. albicans*. The Signature Algorithm [33] was applied to distinguish those genes that are mutually co-expressed in each set [10]. The final set associated with each sequence consisted of the union of co-expressed orthologs from each organism.

### Co-expression of GO terms.

The extent of co-expression of genes assigned to each GO category was quantified by a normalized *t*-value. For each organism, pair-wise Pearson correlation coefficients were evaluated for all gene pairs within the category, using all conditions in the dataset. The resulting distribution was compared to a background distribution of 10,000 randomly chosen gene pairs, and a *t*-statistic was calculated for the two distributions. *t*-Statistics were calculated for all GO categories, as well as for randomly composed control gene sets of the same size distribution. The *t*-values shown in the figure are given in terms of the standard deviation of t-values obtained from the random control sets.

### DCA clustering.

The algorithm was implemented in Matlab using its standard routine for hierarchical clustering with average linkage. The similarity *S_ij_* between genes *i* and *j* was defined by the Euclidean distance between the vectors *C_ik_* and *C_jk_* containing the Pearson correlations (over all experiments) to all the other genes *k*, i.e, 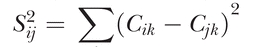


For the HOCA, the Pearson correlations were replaced by the topological overlap, defined below. The cluster definition cutoff was given in terms of the fraction of the maximum linkage value. Cutoff values were chosen heuristically: 0.6 for the gene correlation analysis, 0.4 for the GO term connectivity analysis, and 0.3 for the sequence connectivity analysis.

### Topological overlap.

Following Ravasz et al. [52], the topological overlap between two nodes *i* and *j* in the network was defined as *O_T _*(*i, j*) = *J_n_*(*i, j*)/[min (*k_i_, k_j_*)], where *J_n_*(*i, j*) denotes the number of nodes to which both *i* and *j* are linked (plus 1 if there is a direct link between *i* and *j*), and *k_i_* and *k_j_* are the total number of links of nodes *i* and *j*, respectively.

### Enrichment *p*-values.

Enrichment *p*-values were calculated using the hypergeometric probability density function. The significance *p*-value of observing *z* genes assigned to a given category in a gene set of size *N* is given by 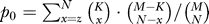 , where *K* is the total number of genes assigned to the category and *M* is the number of genes in the genome. The probability of making a connection between two attributes (GO terms, 6-mers, or 7-mers) is given by 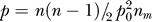 , where *n* is the number of attributes and *n_m_* is the number of representative modules in the dataset (a list of which is given on http://barkai-serv.weizmann.ac.il/candida). Note that this also accounts for multiple hypothesis testing. We imposed a *p*-value of 0.05 for a network connection corresponding to the following significance cutoff for *p_0_* (in units of −log_10_): *C. albicans:* 6-mers: 5.0; 7-mers: 5.6; GO terms: 4.6; *S. cerevisiae:* 6-mers: 4.8, 7-mers: 5.4; GO terms: 4.5.

### Strain construction.

Yeast strain YJB9073 (Figure 5F) was constructed by transforming strain YJB8911 (BWP17 Nop1-CFP) with the PCR amplification product of plasmid pYFP-URA3 [60] and primers F1776 (CAAAAGAAAAAAGAAGAAGAAGAGGATGAGCAAGAAGATGAAGATATTGTAATGGAGGAGGAAGATGATGAGTCTAAAGGTGAAGAATTATT) and R1777 (ATTTAGTCTTGTAT-AACACTATCATATATGTAATATTATTATCGTGTATTAACACAACTGTAAATTATTTGTCTAGAAGGACCACCTTTGATTG), which was designed to insert a *C. albicans* codon-optimized version of YFP at the C-terminus of orf19.5850. The correct integration product was confirmed by PCR with primers F1791 (TTGCAAGCTGTTGATTTCGAACAC) from the middle of *orf19.5850* and R658 (TTTGTACAATTCATCCATACCATG) from the 3′ end of the YFP coding sequence.

## Supporting Information

Figure S1 Illustration of the Use of *t*-Statistics to Evaluate the Extent of Co-Expression of Genes Assigned to a Given Functional Category From left to right: (1) Based on prior functional annotation (as given by the GO or KEGG database), the corresponding subsets of orthologous genes in *S. cerevisiae* and *C. albicans* are selected. (2) Pairwise correlations between these genes are computed in both organisms using the respective set of expression data. (3) The distribution of these correlations are compared to the background distribution corresponding to random subsets of the same size. The significance of co-expression among the functionally associated genes is determined using the *t*-statistics for the two distributions.
(13 KB PDF)

Figure S2 Extent of Co-Expression of Genes Assigned to KEGG Pathways in the Two Organisms Analysis as described for GO terms (c.f. Figure 1A), but using KEGG pathways instead.
(22 KB PDF)

Figure S3 Robustness of Analysis with Respect to Sub-Sampling of Conditions The analysis leading to Figure 3A (left panel) was repeated using only a fraction of the expression data (as indicated above each plot). Note that although the average correlations vary slightly (the error bars denote the standard deviations resulting from different sub-samples), they give rise to the same distinct classifications, even when using only 10% of the available expression data.
(15 KB PDF)

Figure S4 DCA Analysis of Cell-Cycle Genes (Cluster 1) (2.7 MB JPEG)

Figure S5 DCA Analysis of Cell-Cycle Genes (Cluster 2) (2.4 MB JPEG)

Figure S6 DCA Analysis of Cell-Cycle Genes (Cluster 3) (2.0 MB JPEG)

Figure S7 DCA Analysis of Cell-Cycle Genes (Cluster 4) (2.2 MB JPEG)

Figure S8 DCA Analysis of Cell-Cycle Genes (Cluster 5) (2.1 MB JPEG)

Figure S9 DCA Analysis of Cell-Cycle Genes (Cluster 6) (2.0 MB JPEG)

Figure S10 DCA Analysis of Cell-Cycle Genes (Cluster 7) (2.2 MB JPEG)

Figure S11 DCA Analysis of Cell-Cycle Genes (Cluster 8) (2.2 MB JPEG)

Figure S12 DCA Analysis of Cell-Cycle Genes (Cluster 9) (1.7 MB JPEG)

Figure S13 DCA Analysis of Cell-Cycle Genes (Cluster 10) (2.0 MB JPEG)

### 

Interactive versions of Figures S4–S13 are available at http://barkai-serv.weizmann.ac.il/candida/html/cc_analysis.html.

## Abbreviations

DCA: differential clustering algorithm
GO: Gene Ontology
HOCA: higher-order connectivity analysis
ISA: iterative sequence algorithm
ORF: open reading frame
PCM: pair-wise correlation matrix
YFP: yellow fluorescent protein
