# Supplementary figures and images for: Comparative Gene Expression Analysis by a Differential Clustering Approach: Application to the Candida albicans Transcription Program
Source: PLoS Genet. 2005 Sep 30;1(3):e39. doi: 10.1371/journal.pgen.0010039 (PMC1239936; doi:10.1371/journal.pgen.0010039)

GO/KEGG  
functional  
category

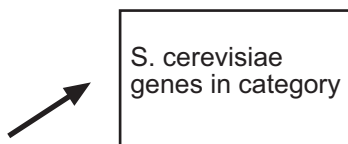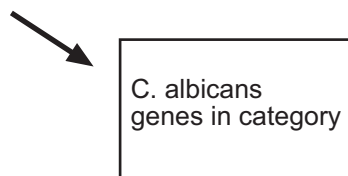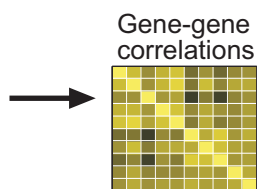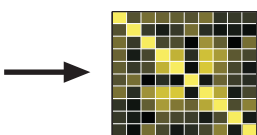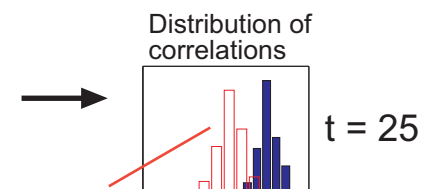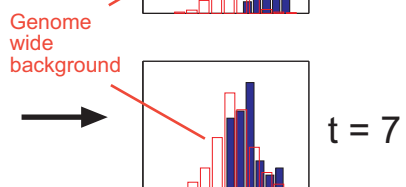

Supplement: Figure S1 — From left to right: (1) Based on prior functional annotation (as given by the GO or KEGG database), the corresponding subsets of orthologous genes in S. cerevisiae and C. albicans are selected. (2) Pairwise correlations between these genes are computed in both organisms using the respective set of expression data. (3) The distribution of these correlations are compared to the background distribution corresponding to random subsets of the same size. The significance of co-expression among the functionally associated genes is determined using the t-statistics for the two distributions. (13 KB PDF) [file pgen.0010039.sg001.pdf]

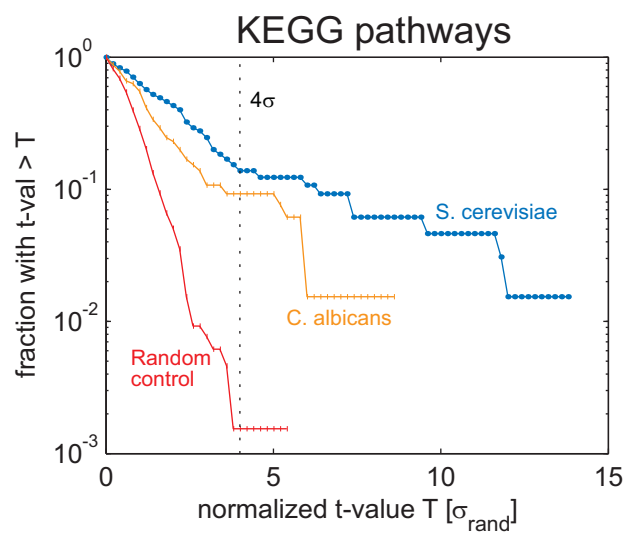

Supplement: Figure S2 — Analysis as described for GO terms (c.f. Figure 1A), but using KEGG pathways instead. (22 KB PDF) [file pgen.0010039.sg002.pdf]

10% of conditions removed

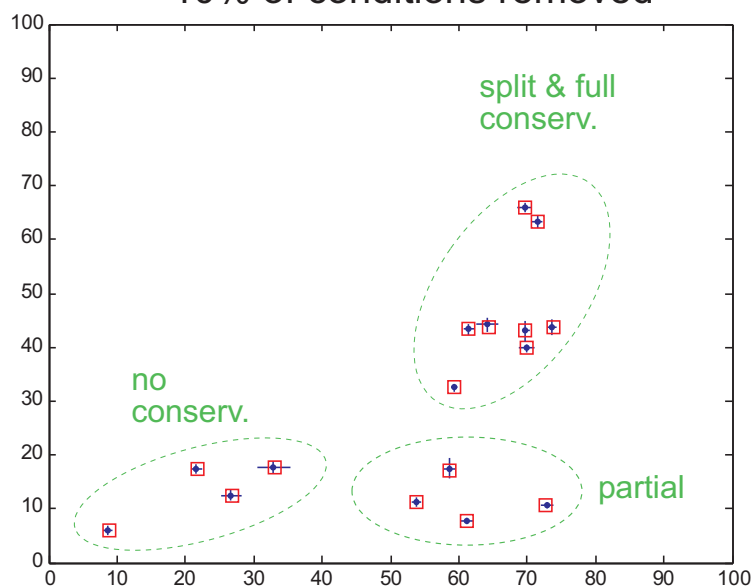

30% of conditions removed

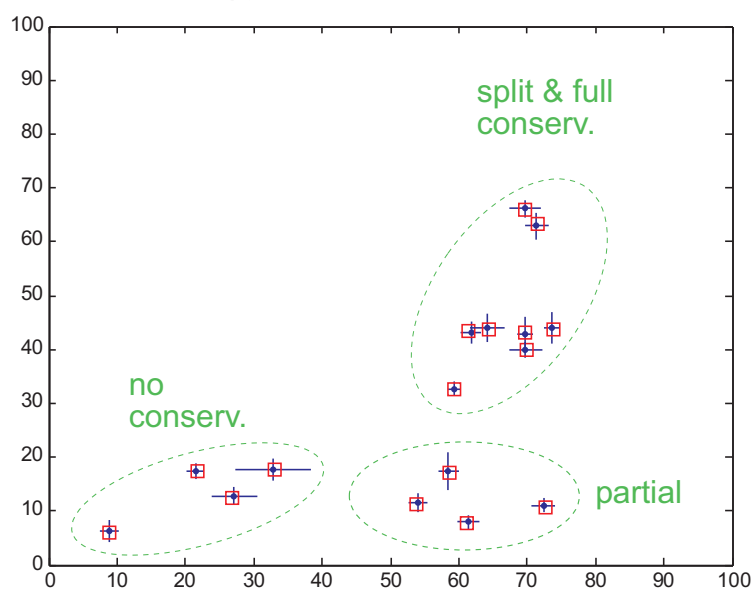

50% of conditions removed

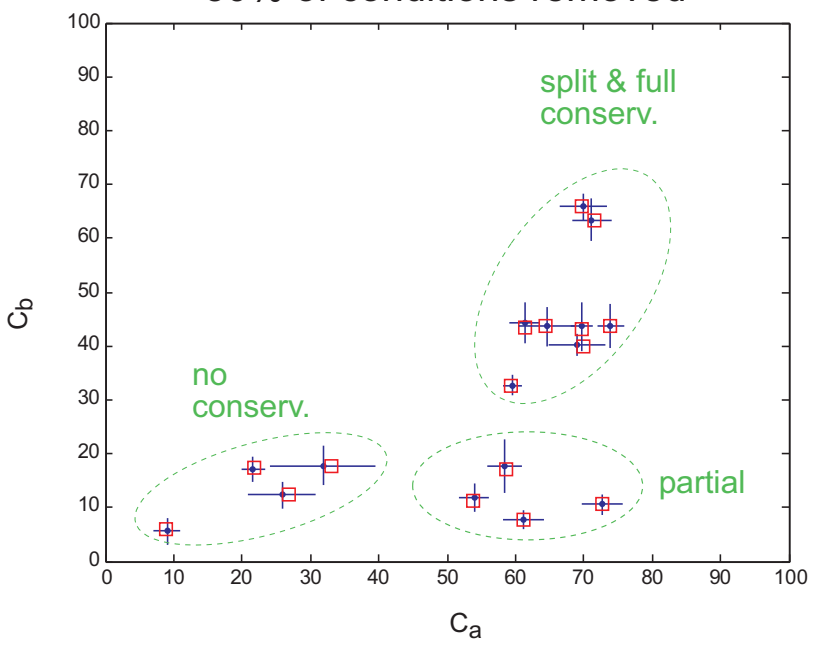

Supplement: Figure S3 — The analysis leading to Figure 3A (left panel) was repeated using only a fraction of the expression data (as indicated above each plot). Note that although the average correlations vary slightly (the error bars denote the standard deviations resulting from different sub-samples), they give rise to the same distinct classifications, even when using only 10% of the available expression data. (15 KB PDF) [file pgen.0010039.sg003.pdf]

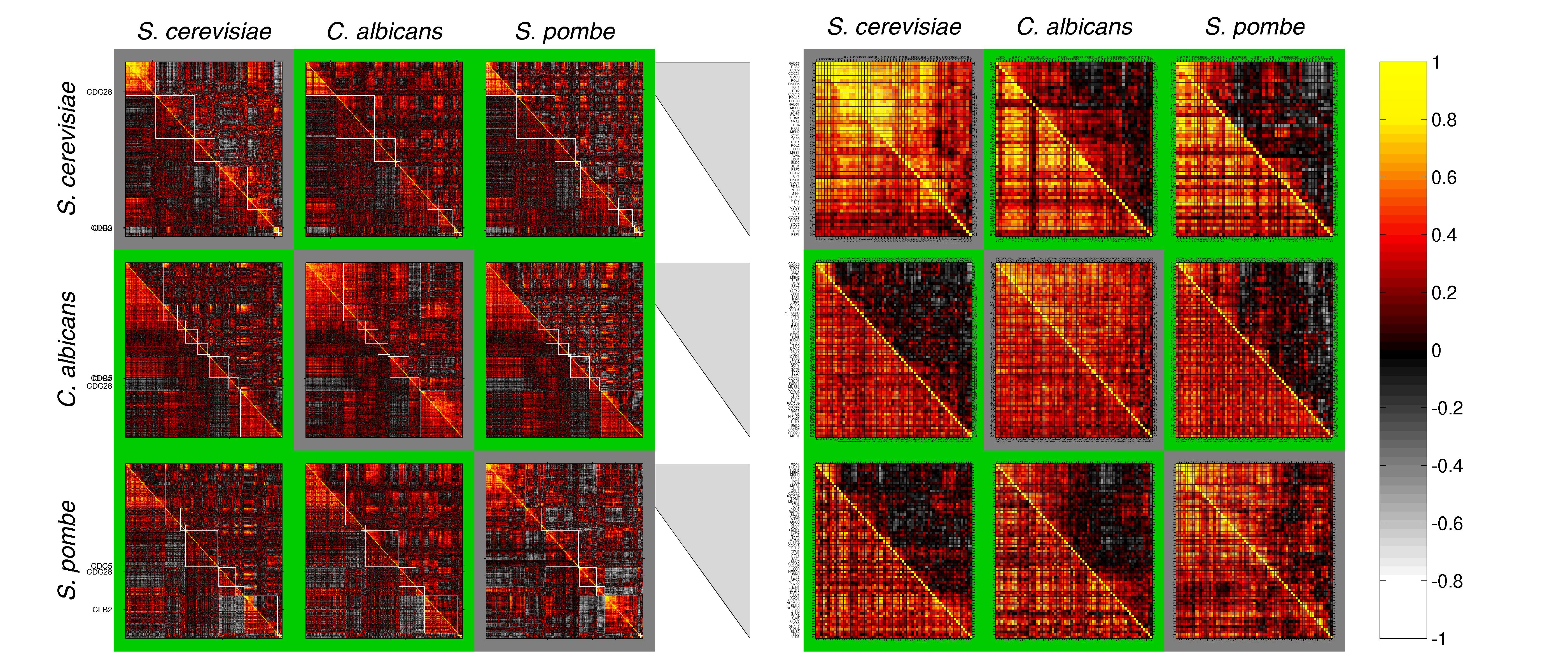

Supplement: Figure S4 — (2.7 MB JPEG) [file pgen.0010039.sg004.jpeg]

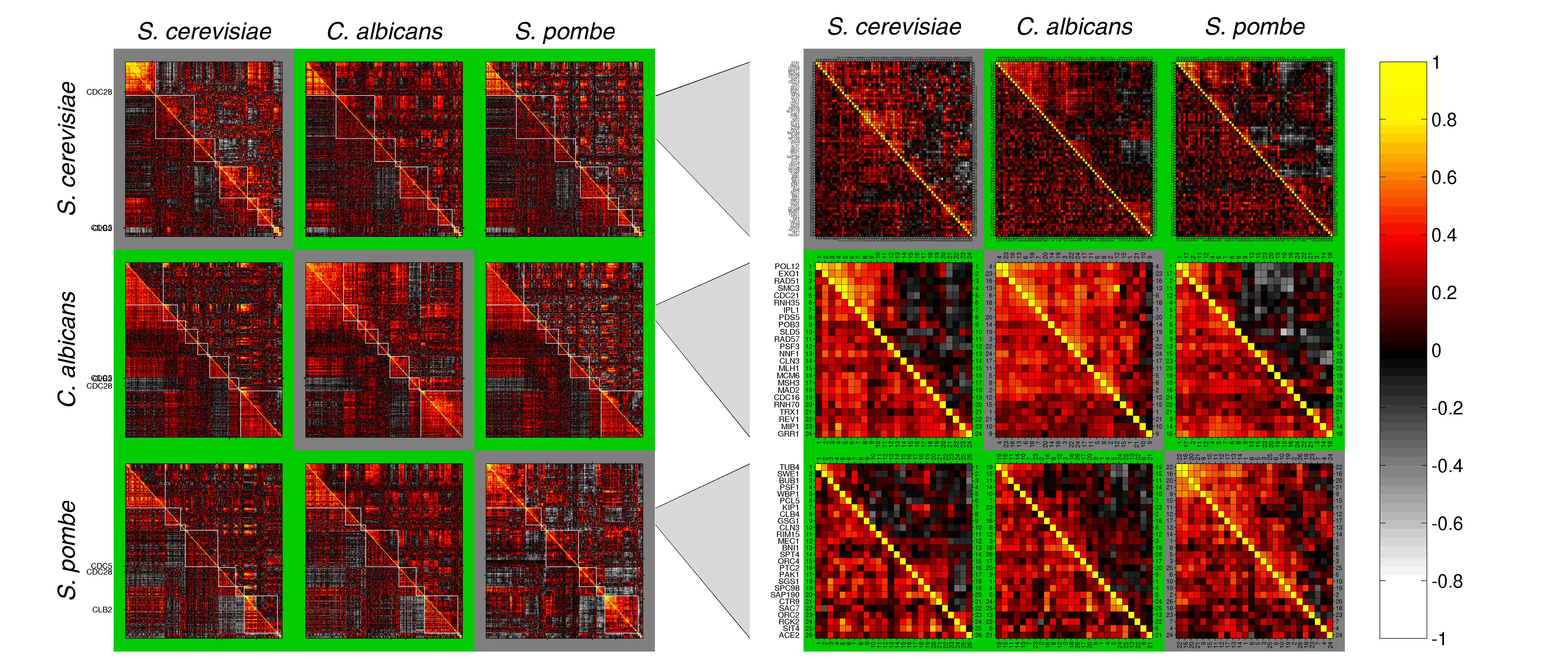

Supplement: Figure S5 — (2.4 MB JPEG) [file pgen.0010039.sg005.jpeg]

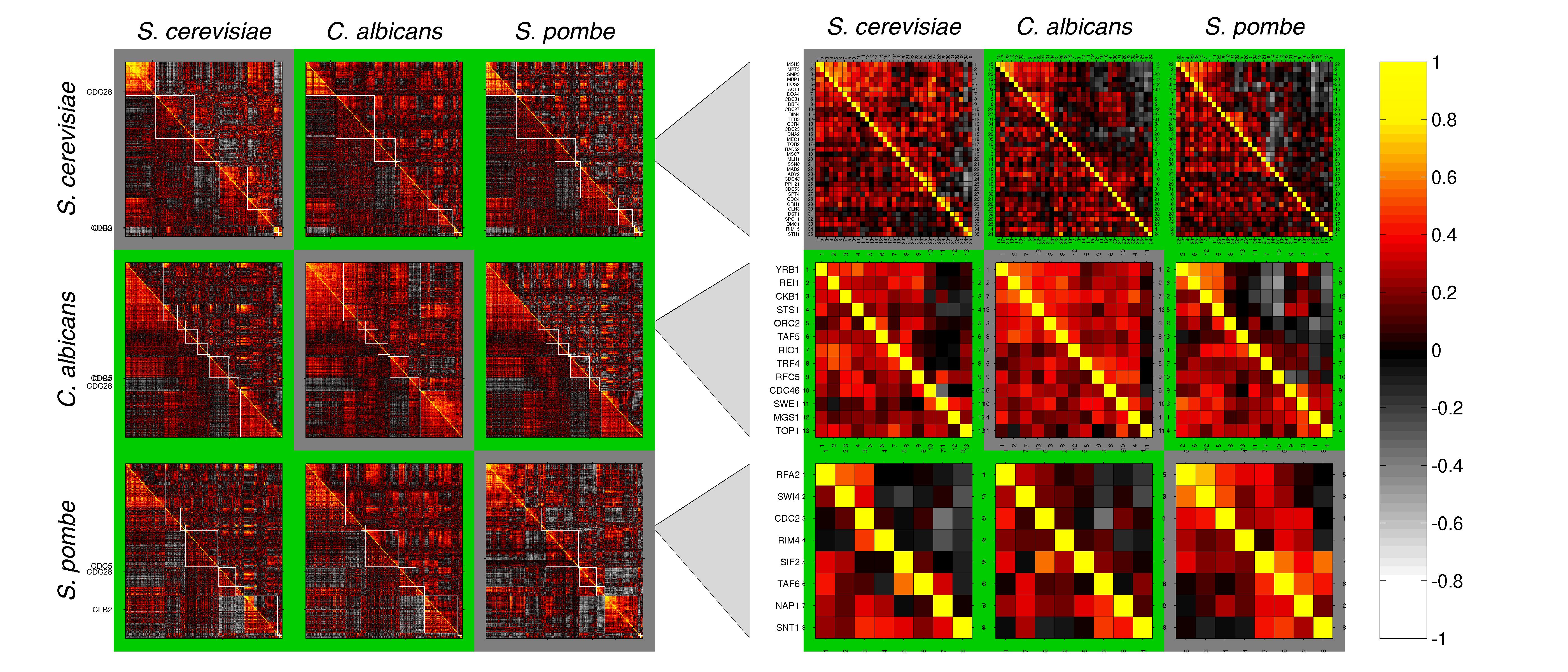

Supplement: Figure S6 — (2.0 MB JPEG) [file pgen.0010039.sg006.jpeg]

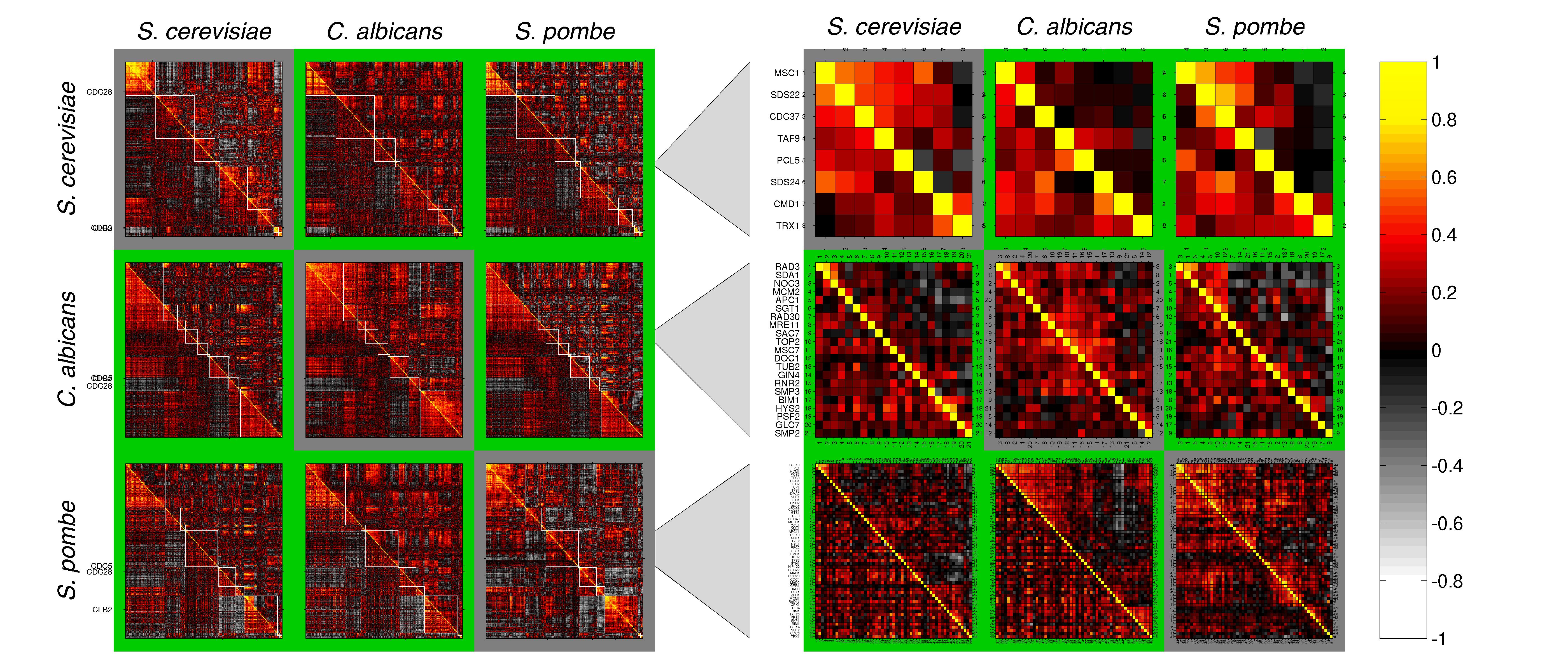

Supplement: Figure S7 — (2.2 MB JPEG) [file pgen.0010039.sg007.jpeg]

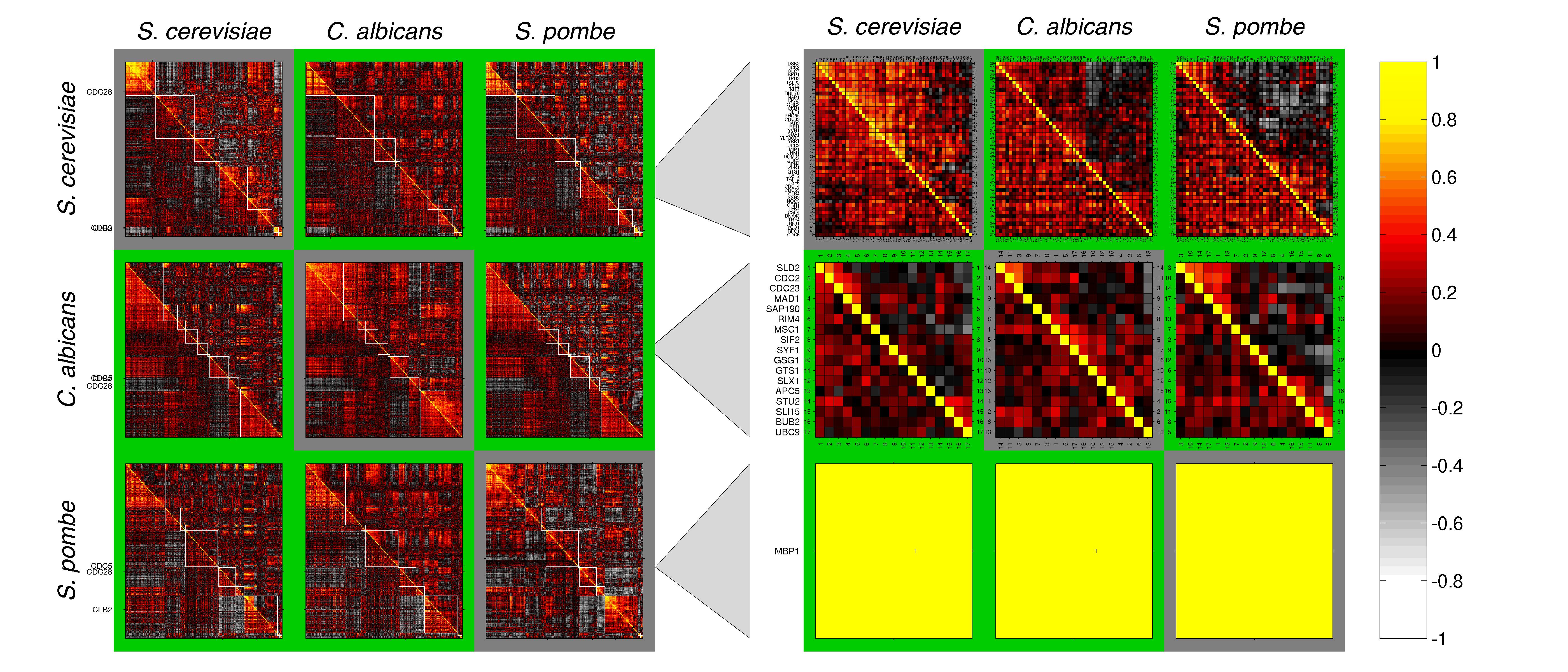

Supplement: Figure S8 — (2.1 MB JPEG) [file pgen.0010039.sg008.jpeg]

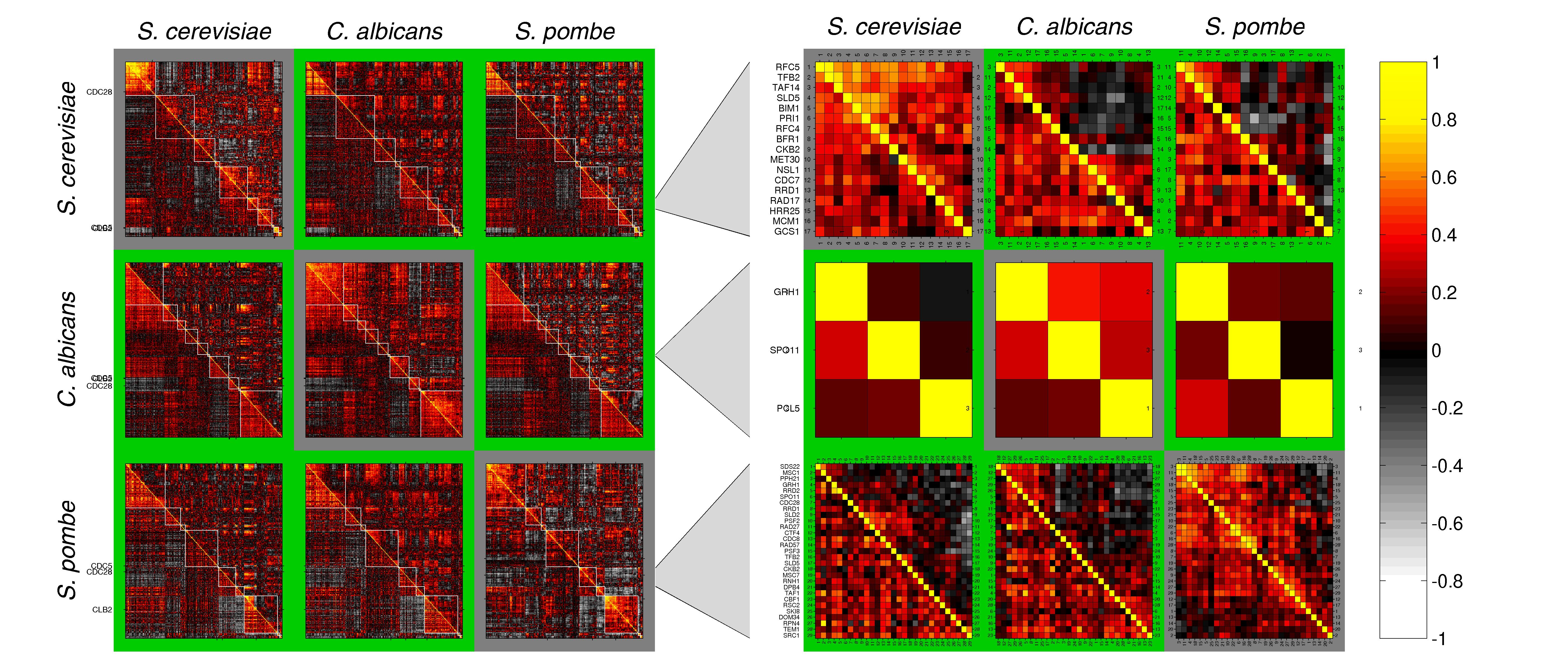

Supplement: Figure S9 — (2.0 MB JPEG) [file pgen.0010039.sg009.jpeg]

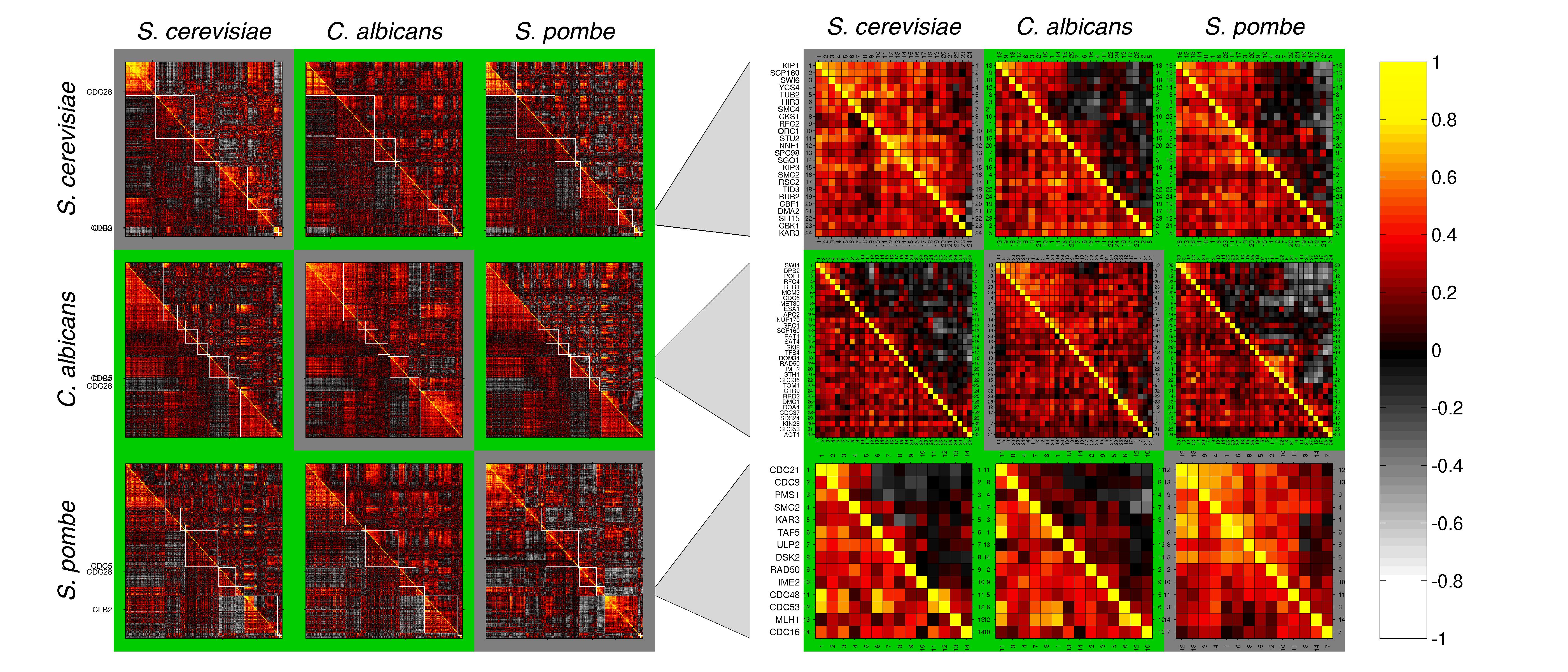

Supplement: Figure S10 — (2.2 MB JPEG) [file pgen.0010039.sg010.jpeg]

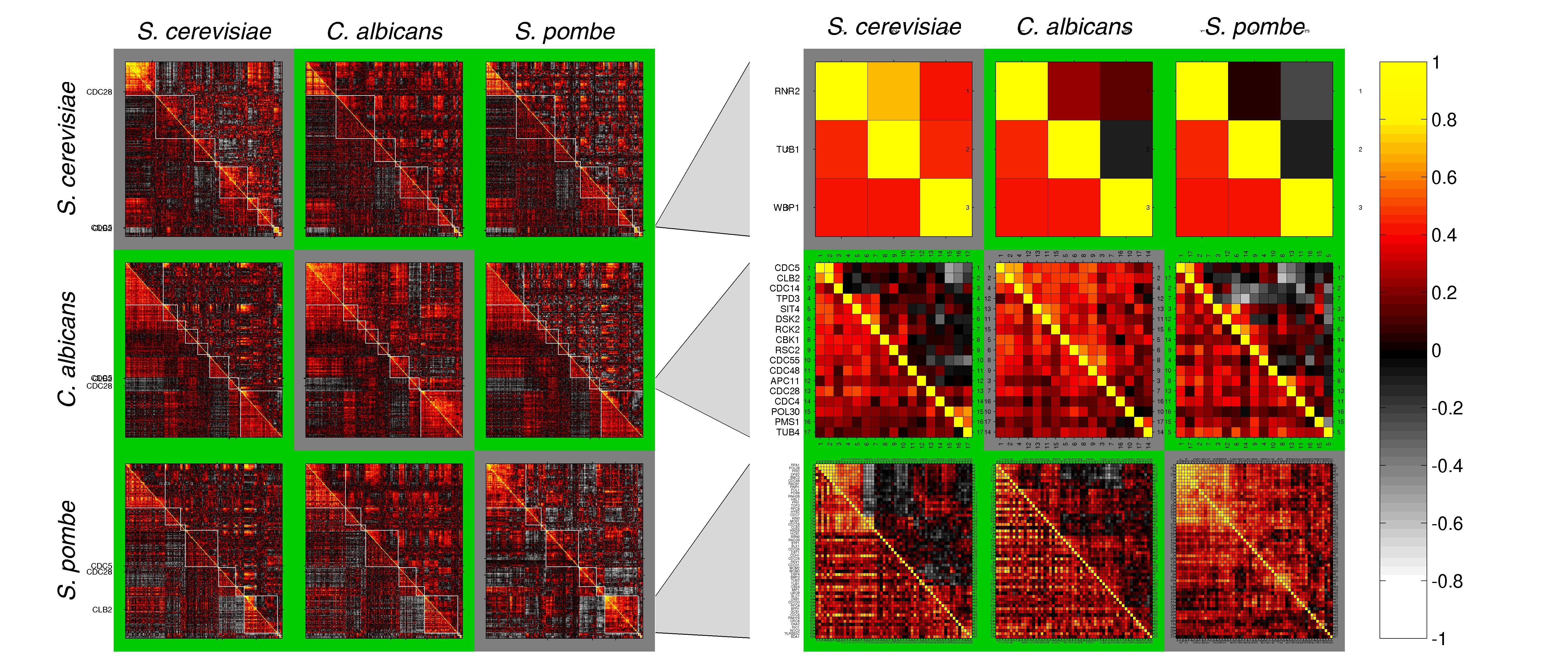

Supplement: Figure S11 — (2.2 MB JPEG) [file pgen.0010039.sg011.jpeg]

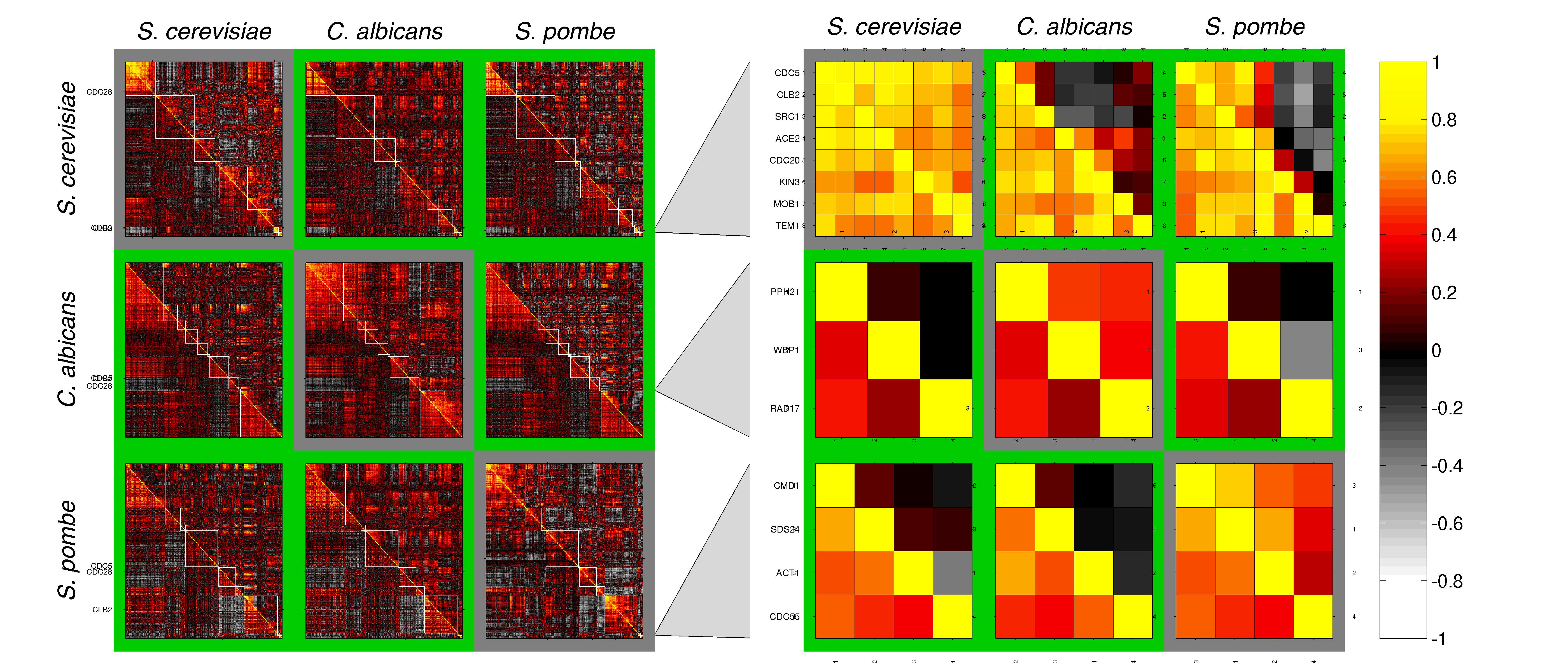

Supplement: Figure S12 — (1.7 MB JPEG) [file pgen.0010039.sg012.jpeg]

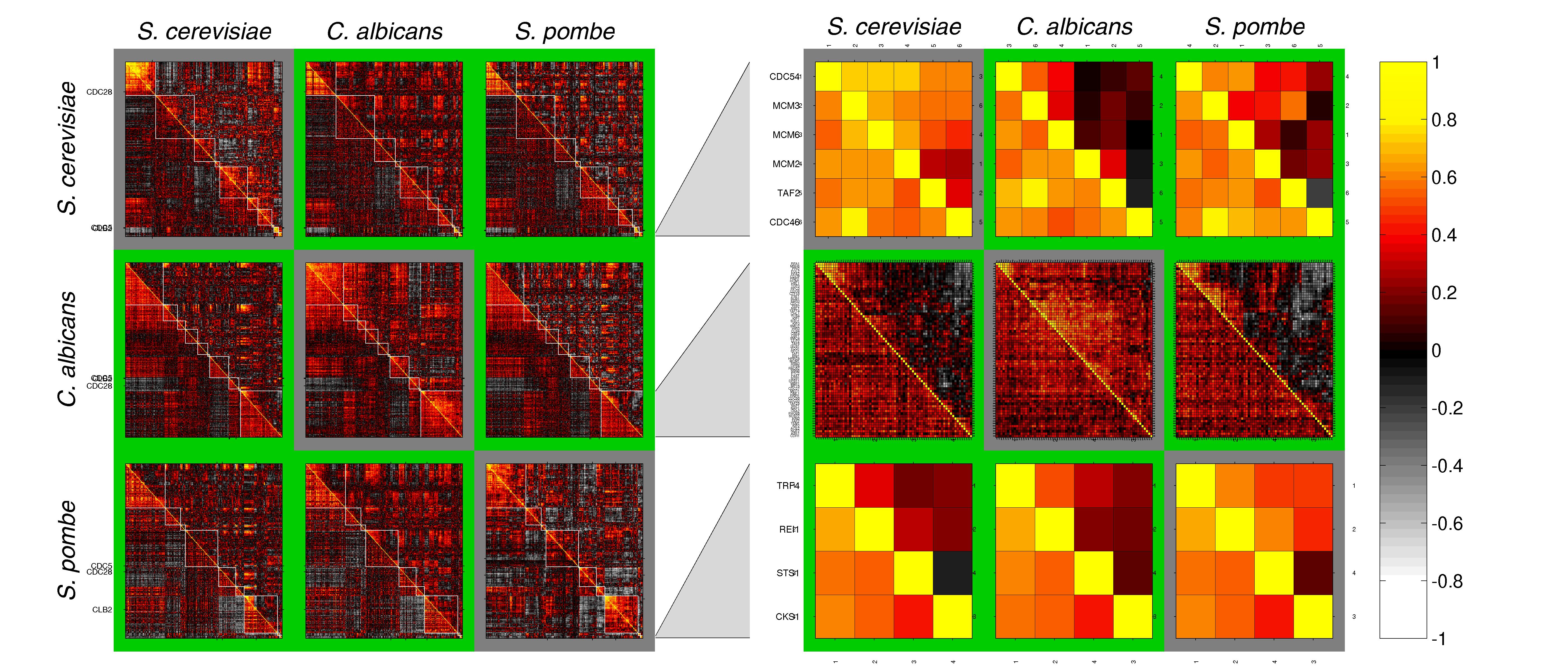

Supplement: Figure S13 — (2.0 MB JPEG) [file pgen.0010039.sg013.jpeg]
